# Editing and Chemical Modifications on Non-Coding RNAs in Cancer: A New Tale with Clinical Significance

**DOI:** 10.3390/ijms22020581

**Published:** 2021-01-08

**Authors:** Ligia I. Torsin, George E. D. Petrescu, Alexandru A. Sabo, Baoqing Chen, Felix M. Brehar, Mihnea P. Dragomir, George A. Calin

**Affiliations:** 1 Department of Anesthesiology and Critical Care, Elias Clinical Emergency Hospital, 011461 Bucharest, Romania; ligiatorsin@gmail.com; 2 Department of Neurosurgery, Carol Davila University of Medicine and Pharmacy, 020021 Bucharest, Romania; gedpetrescu@gmail.com (G.E.D.P.); felix.brehar@umfcd.ro (F.M.B.); 3 Department of Neurosurgery, Bagdasar-Arseni Clinical Emergency Hospital, 041915 Bucharest, Romania; 4 Zentrum für Kinder, Jugend und Frauenmedizin, Pediatrics 2 (General and Special Pediatrics), Klinikum Stuttgart, Olgahospital, 70174 Stuttgart, Germany; saboalexandru@gmail.com; 5 State Key Laboratory of Oncology in South China, Department of Radiation Oncology, Collaborative Innovation Center of Cancer Medicine, Sun Yat-sen University Cancer Center, Guangzhou 510060, China; chenbq@sysucc.org.cn; 6 Guangdong Esophageal Cancer Research Institute, Guangzhou 510060, China; 7 Institute of Pathology, Charité-Universitätsmedizin Berlin, 10117 Berlin, Germany; 8 Department of Translational Molecular Pathology, The University of Texas MD Anderson Cancer Center, Houston, TX 77030, USA; 9 Center for RNA Interference and Non-Coding RNAs, The University of Texas MD Anderson Cancer Center, Houston, TX 77054, USA

**Keywords:** RNA editing, RNA chemical modifications, cancer, non-coding RNA, microRNA, long non-coding RNA, circular RNA

## Abstract

Currently, for seemingly every type of cancer, dysregulated levels of non-coding RNAs (ncRNAs) are reported and non-coding transcripts are expected to be the next class of diagnostic and therapeutic tools in oncology. Recently, alterations to the ncRNAs transcriptome have emerged as a novel hallmark of cancer. Historically, ncRNAs were characterized mainly as regulators and little attention was paid to the mechanisms that regulate them. The role of modifications, which can control the function of ncRNAs post-transcriptionally, only recently began to emerge. Typically, these modifications can be divided into reversible (i.e., chemical modifications: m^5^C, hm^5^C, m^6^A, m^1^A, and pseudouridine) and non-reversible (i.e., editing: ADAR dependent, APOBEC dependent and ADAR/APOBEC independent). The first research papers showed that levels of these modifications are altered in cancer and can be part of the tumorigenic process. Hence, the aim of this review paper is to describe the most common regulatory modifications (editing and chemical modifications) of the traditionally considered “non-functional” ncRNAs (i.e., microRNAs, long non-coding RNAs and circular RNAs) in the context of malignant disease. We consider that only by understanding this extra regulatory layer it is possible to translate the knowledge about ncRNAs and their modifications into clinical practice.

## 1. Introduction

After the sequencing of the human genome, when it was discovered that the protein coding DNA account for little over 1% and the majority of the DNA is non-coding, one of the greatest adventures of molecular biology started, namely deciphering the role of “junk DNA” [1]. An opening answer came quickly thereafter, when it was discovered that a significant part of the non-coding DNA is pervasively transcribed into non-coding RNA (ncRNA) [2]. Nowadays, it is well known that the number of non-coding genes surpasses that of coding-genes and the roles of ncRNAs in physiology and pathology are recognized [3,4,5].

MicroRNAs (miRNAs) are small regulatory RNAs, of approximately 22 nucleotides, which interact with target mRNAs to induce mRNA degradation and translational repression. MiRNAs are transcribed from DNA sequences into primary miRNAs (pri-miRNA) and processed by the ribonuclease type III Drosha into precursor miRNAs (pre-miRNA), and further processed by Dicer into mature miRNAs [6,7]. For a comprehensive description of miRNA biogenesis and function, please refer to the review by Hausser and Zavolan [8]. Long non-coding RNAs (lncRNAs), on the other hand, are usually defined as transcripts with lengths exceeding 200 nucleotides that share some similarities with mRNAs but do not encode proteins [9,10]. Circular RNAs (circRNAs) are closed RNA loops, in which the 5′ and 3′ termini are covalently linked by back-splicing of exons from a single pre-mRNA [11].

In the last decades, after the first ncRNA molecules were linked to cancer [12], we have witnessed an explosion of studies that suggest the implication of ncRNAs in the malignant disease [13,14,15,16,17]. At this point, for every type of cancer, dysregulated levels of ncRNAs were reported and non-coding transcripts are expected to be the next class of diagnostic and therapeutic tools in oncology [3,18,19].

NcRNAs were characterized as regulators and little attention was paid to the mechanisms that regulate them. To some extent, the regulatory modifications that control the ncRNAs transcription at the DNA level were partially described and are reviewed elsewhere [20], but the modifications at the RNA level, that control the function of ncRNAs post-transcriptionally, have only recently begun to emerge. Typically, the modifications at the ncRNA level can be divided into two categories, reversible (i.e., chemical modifications) and non-reversible (i.e., editing) [21]. These modifications have been studied in the past decades mostly in mRNA, and with a brief focus on some “functional” ncRNAs such as transfer RNAs (tRNAs) and ribosomal RNAs (rRNAs). One of the first articles describing ncRNA modifications in cancer appeared in 2012, when Squires et al. showed for the first time that the modified 5-methylcytosine (m^5^C) is widespread in ncRNA transcripts of HeLa cells, indicating a broader function of this chemical modification in malignancy [22]. Hence, the aim of this review paper is to describe the most common regulatory modifications (editing and chemical modifications) of the most studied species of traditionally considered “non-functional” ncRNAs in the context of malignant disease and to hypothesize how these could aid the implementation of ncRNAs as diagnostic and therapeutic tools. Moreover, if the regulatory modifications were described only in tRNAs and rRNAs, we hypothesize that these alterations could also exist in miRNAs, lncRNAs, and circRNAs.

## 2. NcRNA Editing in Cancer

RNA editing refers to alterations of the RNA’s nucleotide sequence, specified in the genomic template, by either insertion, deletion or addition of non-template nucleotides or base conversion through amination or deamination processes. These co-transcriptional and post-transcriptional modifications influence the biogenesis and the functions of RNAs, including ncRNA, which have been associated with the onset of human diseases, including cancer [23]. The development of next-generation sequencing technologies, together with bioinformatics tools, led to the genome-wide detection of numerous ncRNAs modifications [24,25,26,27].

The most common editing event is by far adenosine to inosine (A-to-I) transformation, catalyzed by adenosine deaminase acting on RNA (ADAR) enzymes. The second in line is cytidine to uridine (C-to-U) editing, which is controlled in humans by a cytidine deaminase named apolipoprotein B mRNA editing enzyme, catalytic polypeptide-like (APOBEC) [28]. More recently, non-ADAR, non-APOBEC ncRNA editing mechanisms have also been described [29].

### 2.1. ADAR Dependent Editing

ADAR1, expressed in almost all tissues, and ADAR2, highly expressed in brain, are the two enzymes that act on RNA by deamination of the sixth carbon of adenosine that leads to inosine formation. Inosine pairs preferentially with cytidine, being thus interpreted as guanosine by the translational machinery [30]. Furthermore, the ADAR family also contains ADAR3, a protein that does not have enzymatic proprieties, is expressed only in brain cells, and negatively correlates with RNA A-to-I editing levels. ADAR3 acts as an inhibitor of editing by competing with the active ADARs for binding the substrates [31].

ADARs can edit the primary structure of either pri-miRNA or pre-miRNA, at the level of Drosha/Dicer recognition sites or close to them, which can lead to an impaired processing and decreased levels of mature miRNAs [32], but editing events that promote Dicer cleavage were also described [33]. Furthermore, an editing event corresponding to the mature sequence of the miRNA can either decrease the mature miRNA expression because of steric binding inhibition of the ribonucleases, or lead to target redirection [28]. By systematically analyzing miRNAs across 20 cancer types, Wang et al. discovered 19 unique A-to-I RNA editing hotspots that have extensive correlations with clinical variables, such as tumor stage or patient survival [34]. Another systematic analysis of 32 cancer types and normal controls identified 58 miRNA editing sites, the majority in the seed regions of miRNAs [35]. Both hyper-editing and hypo-editing events were linked to malignancy. For instance, in a series of breast, lung, ovarian and renal cancer cell lines, the lack of miR-379-5p editing leads to cancer cell proliferation and inhibition of apoptosis. Under normal circumstances A-to-I editing of miR-379-5p reduces the expression of CD97, at both mRNA and protein level [36]. CD97 is up-regulated in multiple types of cancer and its overexpression is clinically correlated with worse patient survival [37,38,39].

In the brain, ADAR2 edits a large number on miRNAs, most of which act as oncogenic miRNAs (onco-miRNAs), consequently reducing their expression. The impairment of ADAR2 activity in glioblastoma leads to non-edited miR-222/221 and miR-21 precursors and increased expression levels of the corresponding mature onco-miRNAs, thus inducing cell proliferation and migration [40]. Moreover, Tomaselli et al. also underlined that in glioblastoma cells ADAR2 may control miRNA expression not only by editing miRNA precursors directly, but also by editing other RNAs involved in the process of their maturation. Thus, although ADAR2 can edit 19 specific sites of 18 miRNAs, its inactivation correlates to the down-regulation of 60 miRNAs and the up-regulation of other 31 miRNAs in glioblastoma cells [40].

In another glioblastoma study, unedited miR-376a becomes unable to target the mRNA of the autocrine motility factor receptor (AMFR) and redirects the target to RAP2A, a member of the RAS oncogene family. Consequently, RAP2A is down-regulated, inducing invasiveness, and AMFR is up-regulated, promoting cancer invasion and migration [41]. Editing at the fifth position of the mature miR-200b-3p impairs the ability to inhibit ZEB1/ZEB2 and acquires a new target-leukemia inhibitory factor receptor (LIFR)-conferring a net effect of increasing motility and invasion in a series of cancer cell lines [34]. Another example of mRNA retargeting in glioblastoma is the unedited miR-589–3p that redirects its target from the tumor suppressor PCDH9 to ADAM12, a metalloproteinase that promotes cancer invasion [42].

**Figure 1:**
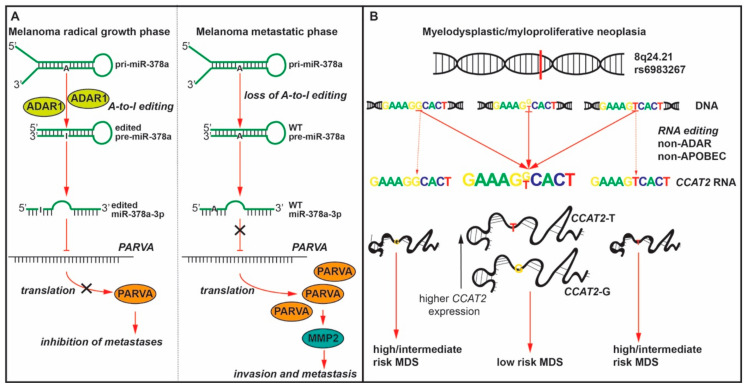
Two typical examples of ncRNA editing. (**A**) In the early phase of melanoma development (melanoma radical growth phase) an ADAR1 dependent editing mechanism protects against metastasis. The pri-miR-378a transcript suffers an A-to-I editing which enables the mature miR-378a-3p to bind the *PARVA* (*parvin alpha*) mRNA and inhibit its translation, blocking this pro-metastatic mechanism. In the late phase of melanoma development (melanoma metastatic development) the expression of ADAR1 is reduced and the A-to-I editing is lacking. The wild-type (WT) miR-378a-3p is not inhibiting the translation of *PARVA*, which is accumulating and inducing invasion and metastasis via MMP2 (matrix metallopeptidase 2). (**B**) *CCAT2* gene is located in the 8q24 region and its transcript contains the single nucleotide polymorphism (SNP) G/T rs6983267. The G allele of the SNP is associated with increased risks for multiple types of cancer. Recently, it was shown that a non-ADAR, non-APOBEC editing mechanism can generate both *CCAT2*-G and *CCAT2*-T transcripts although the DNA is homozygote for G or T. Clinically, these heterozygote pool of *CCAT2* transcripts is associated with low risk myelodysplastic syndrome (MDS).

Under the influence of transcription factor cAMP-response element binding protein (CREB), ADAR1 expression decreases with melanoma progression, which decreases A-to-I RNA editing of miR-378a-3p, miR-324-5p, and miR-455-5p. The unedited miR-455-5p down-regulates the tumor suppressor gene cytoplasmic polyadenylation element-binding protein 1 (CPEB1), while edited miR-455-5p targets and down-regulates RHO-C, MDM4, and integrin α2, all of which have known tumor promoting functions [43]. Thus, the lack of miRNA editing leads to melanoma growth and metastasis. Similarly, the edited form of miR-378a-3p binds to the mRNA of parvin alpha (*PARVA*) and inhibits its expression. *PARVA* is an oncogene that plays a role in cell adhesion, motility, and survival. In non-metastatic melanoma cells, miR-378a-3p is subjected to A-to-I editing, but not in the metastatic cells. This leads to an increased expression of PARVA and to cell invasion (Figure 1A), probably through the degradation of extracellular matrix [44].

ADAR2-mediated editing of the complementary antisense transcripts of pri-miR-214 induces an unusual U-to-C change of pri-miR-214, attributed to the A-to-I editing on the complementary transcripts of pri-miR-214. Thus, it disrupts the maturation of miR-214, which results in an increased expression of a RAS family oncogene RAB15 [45]. A similar regulation was observed in leukemia stem cells, where ADAR1 promotes self-renewal gene expression via let-7 pri-miRNA editing and LIN28B up-regulation [46].

In non-small-cell lung cancer (NSCLC) it was discovered that ADAR1 is frequently amplified and plays a tumorigenic role, being associated with shorter overall survival [47]. Mechanistically, the high levels of ADAR1 increase A-to-I editing of miR-381-3p, a tumor suppressor miRNA that inhibits NF-kB signaling [48], as well as the editing of the DNA repair enzyme Endonuclease 8-like 1 (NEIL1) [47]. Further studies are necessary to detect the downstream targets of the edited miR-381-3p.

Moreover, A-to-I RNA editing events in the 3′ untranslated region (3′ UTR) of mRNAs can alter miRNA-mRNA interaction. For example, Rho GTPase activating protein 26 (*ARHGAP26*) mRNA normally undergoes extensive A-to-I RNA editing in the 3′ UTR that is catalyzed by ADAR1 [49]. ARHGAP26 is a Rho GTPase-activating protein that has a tumor suppressor role. If *ARHGAP26* mRNA does not undergo A-to-I editing, the 3′ UTR pairs with miR-30b-3p and miR-573, inhibiting its translation. This leads to an enhanced activity of RhoA and Cdc42 proteins, known for growth-promoting effects malignant transformation [49]. Another example is the editing of the 3′ UTR of mRNA of DNA fragmentation factor subunit alpha (*DFFA*), a protein that triggers DNA fragmentation during apoptosis. ADAR1 editing of *DFFA* mRNA in a cell line of non-invasive, hormone-responsive type of breast cancer renders the mRNA unrecognizable by miR-140-3p, thus increasing the levels of DFFA and inducing apoptosis, while the lack of editing in a cell line of highly invasive, triple-negative breast cancer disallows DFFA regulation by miR-140-3p [50]. Pinto et al. found across 9 different types of cancers over 63,000 editing sites within the 3′UTRs of mRNAs, rich in Alu elements, which function as recruitment elements for ADAR. These editing events can either create a novel target for a miRNA or simply destroy the complementarity between a miRNA and the mRNA [35].

To add yet another layer of complexity, in hepatocellular carcinoma (HCC) cells, ADARs were found to directly bind to Dicer and, without an editing event, this leads to an augmentation of the processing of pre-miR-27a to mature miR-27a. MiR-27a binds to 3′ UTR of methyltransferase like 7A (*METTL7A*), a known tumor suppressor, decreasing its expression [51]. Furthermore, ADAR1 can regulate miRNA processing in an RNA binding–dependent, but editing–independent manner. ADAR1 can regulate Dicer expression at the translational level via the lethal-7 (let-7) gene, thus indirectly affecting miRNA biogenesis. ADAR1 can also directly interact with miRNA maturation by competitively binding to DGCR8 and affecting the formation of DGCR8/Drosha cleavage complex [52].

### 2.2. APOBEC Dependent Editing

Unlike A-to-I editing, C-to-U editing is less common. There are 11 cytidine deaminases expressed in humans, each family member with a different tissue specificity and subcellular localization [53]. APOBEC misregulation has been linked to cancer progression, prognosis and response to therapy [54,55,56,57]. APOBEC family can interact with miRNA function either through editing-dependent or independent mechanisms. Although there are no studies that demonstrate a direct C-to-U editing event in miRNAs, APOBEC’s editing of 3′ UTRs of mRNAs may be able to change miRNAs recognition sequence by either creating or eliminating seed motifs [58]. APOBEC1 editing is site-specific and needs a cytidine located in AU-rich regions and a specific mooring sequence motif within the edited RNA sequence. Moreover, APOBEC1 requires an RNA-binding protein cofactor, either A1 complementation factor (ACF) or RBM47 [59]. Liver-specific over-expression of APOBEC1 in transgenic mice or rabbits leads to aberrant editing of hepatic mRNA and is correlated with HCC development [60]. In an animal model of lung cancer, APOBEC1 levels showed a 1.5-fold increase, while 16 sites in the 3′UTR of transcript RNA showed increased levels of C-to-U editing. Although the editing did not occur in tumor-originating cells, it is possible that C-to-U editing in surrounding cells may contribute to cancer progression [61].

APOBEC3 family members inhibit the activity of a wide range of endogenous retro-elements, such as long terminal repeats (LTRs) and non-LTRs, as well as exogenous retro-elements [62,63]. Although the exact mechanism is not known, it was shown that APOBEC3G overexpression correlates with the up-regulation of miR-205, miR-212, miR-126, and miR-181c, and down-regulation of miR-29a in colon cancer cells. Furthermore, by hampering miR-29a activity in repressing matrix metallopeptidase 2 (MMP2), APOBEC3G promotes colorectal cancer (CRC) liver metastasis [64].

Moreover, APOBEC3 can regulate miRNA activity through the inhibition of dead-end protein homolog 1 (DND1). DND1 is a protein that regulates the activity of miRNAs by blocking their interaction with the 3′ UTR of specific mRNAs, and thus restoring protein expression. APOBEC3G is able to block DND1 function, but the exact mechanism is not known. It may be either through binding or sequestration of DND1, permitting the interaction of miRNAs with their target mRNA [65]. Thus, miR-221, miR-372, and miR-206 can inhibit the translations of *P27*, *LATS2*, and *CX43*, respectively, all of which are implicated in the pathology of cancer [66,67,68]. The second hypothesis is that APOBEC3G binds to the mRNAs and interacts with miRNA-induced silencing complex (miRISC), activating translation repression [65]. Another possibility is that APOBEC3G edits the 3′ UTR of mRNAs, thus inhibiting DND1 binding [65].

### 2.3. Non-ADAR, Non-APOBEC Editing Mechanisms

It was shown that the differences between the RNA sequence and DNA sequence are much more common than thought and that other mechanisms of editing than those mediated by ADARs and APOBECs exist. This hypothesis was launched by a group from University of Michigan led by Vivian Cheung, which discovered that there are over 10,000 sites where the coding RNA does not match the DNA and that these modified mRNAs are translated into peptides that do not match the DNA sequence. The authors discovered that there are 12 possible nucleotide substitutions between DNA and RNA, therefore other mechanisms that can explain these changes need to be elucidated. Additionally, the authors state that these widespread editing mechanisms are shared by different cells, including tumor cells (neuroblastoma and lung carcinoma) [69]. Subsequent studies examined these findings with caution, even claiming that more than 88% of the RNA-DNA differences (RDD) could be simple technical artefacts [70,71,72]. In a following research article, the group led by Vivian Cheung showed that RDDs take place immediately after transcription (55 nucleotides away from RNA Polymerase II) and suggest that this phenomenon is independent of the RNA editing enzymes and could be linked to R-loop formation [73].

More recently, our group showed that at the site of single nucleotide polymorphism (SNP) rs6983267 (G/T) a similar phenomenon occurs. The rs6983267 SNP is transcribed as part of a 1.7-kb lncRNA termed *CCAT2*, which was proved to play an oncogenic role in CRC [74] and induce chromosomal instability via BOP1–Aurora Kinase B pathway [75]. Epidemiologic data demonstrated that the G allele of this SNP increases the risks for multiple types of cancers including colon, breast, prostate, and bladder cancers [76,77,78,79]. We also discovered that the G allele of *CCAT2* preferentially induces the splicing of the glutaminase isoform C (GAC) of glutaminase (GLS). The GAC isoform is the more active one and is partially responsible for increased cell proliferation and metastases of *CCAT2*-G tumors [80]. Using a *CCAT2* transgenic mouse model (*CCAT2*-G or *CCAT2*-T) we showed that the overexpression of this lncRNA induces in vivo myelodysplastic/myeloproliferative neoplasms (MDS/MPN). Additionally, we observed that *CCAT2* is overexpressed in peripheral blood cells and in the bone marrow of patients with MDS/MPN. Surprisingly, by using two independent methods (allele-specific restriction enzyme digestion and Sanger sequencing) we detected that the complementary DNA (cDNA) does not match the genomic DNA (gDNA) at the SNP locus in MDS/MPN patients, and the phenomenon was termed DNA-to-RNA allelic imbalance (DRAI). The number of heterozygote patients at the gDNA level was not matching the number of heterozygote patients at the cDNA level and the level of heterozygosity was always increasing at the RNA level. It is important to remark that a heterozygote genotype/pool of transcripts (*CCAT2*-G/T) is associated with low risk MDS (Figure 1B). In vivo studies confirmed these findings. Although all mice were homozygotes at the gDNA level (GG or TT), at the cDNA level, 34% of the mice were heterozygotes (*CCAT2*-G/T). Phenotypically, heterozygote mice had more often splenomegaly, hepatomegaly, and bone marrow hyper-cellularity [29].

In conclusion, it also seems that other mechanisms of editing than ADAR and APOBEC-dependent exist, and they not only affect the coding RNA. A list of ncRNA editing phenomena is presented in Table 1. These editing mechanisms are altered in pathological states and may induce the development of and modify the course of neoplastic disease. Future studies are highly necessary to determine how widespread they are and if they are systemic, purposeful, and regulated.

## 3. NcRNA Chemical Modifications in Cancer

**Figure 2:**
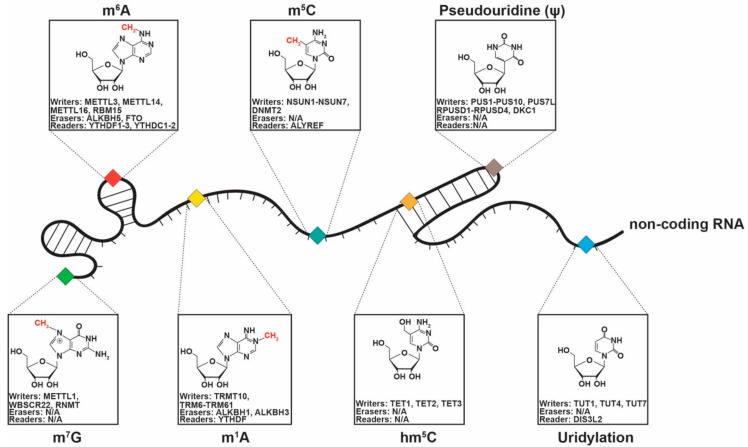
Chemical modifications (m^7^G = 7-Methylguanosine, m^6^A = N^6^-Methyladenosine, m^1^A = N^1^-Methyladenosine, m^5^C = 5-methylcytosine, hm^5^C = 5-hydroxymethylcytosine, Pseudouridylation (Ψ), and Uridylation) described in ncRNAs, including lncRNAs, miRNAs, tRNAs, rRNAs and their specific writers, erasers, and readers.

In the past years we have witnessed an explosion of papers describing chemical modifications of mRNAs [81]. Only more recently were some of these chemical modifications described also in traditionally considered “non-functional” ncRNAs (miRNAs, lncRNAs, and circRNAs) and the better characterized “functional” ncRNAs (rRNAs, and tRNAs) (Figure 2), and a few of them were shown to play a role in cancer pathogenesis. Therefore, in this section, we will present the role of the most common chemical modifications in ncRNAs and their link to cancer.

### 3.1. 5-Methylcytosine

m^5^C was initially considered to be present only in tRNAs and rRNA [21]. Recent evidence confirms that m^5^C also exists in mRNAs, viral RNAs [82] and ncRNAs [83]. Two categories of m^5^C writers are responsible for the chemical modifications [84]: NOP2/Sun RNA methyltransferases (NSUNs) family and DNA methyltransferases (DNMTs). The NSUN family consists of seven members, among which the overexpression of NSUN2 has been identified in cancer [85,86] and is associated with metastasis in breast cancer [87]. Also, NSUN1 is marker for tumorigenesis [88]. The overexpression of NSUN1 (p120) is associated with an unfavorable prognosis in prostate cancer and lung adenocarcinoma [89,90]. Additionally, dysregulated levels of DNMT2 have been identified in cancer cells [91]. DNMT2 has been shown to be up-regulated in prostate carcinoma cell from patients who underwent treatment [92]. ALYREF mRNA export adapter is the only reader described to date for m^5^C [93]. No erasers have been identified for m^5^C, but ten-eleven family demethylases (TET) can turn m^5^C into 5-hydroxymethylcytosine (hm^5^C) in vitro [94].

Konno et al. demonstrated that several types of RNA methyltransferases are up-regulated in pancreatic cancer and CRC. The authors transfected m^5^C modified and N^6^-Methyladenosine (m^6^A) modified miR-200c-3p in HCT116 Dicer deficient cells and observed that only m^5^C miR-200c-3p can reduce the expression level of its target mRNAs. Next, by using a novel method to detect miRNA methylation, matrix-assisted laser desorption/ionization-time of flight-mass spectrometry (MALDI-TOF-MS), they discovered that the levels of methylated miR-200c-3p, miR-21-3p (both m^5^C modified), let-7a-5p, and miR-17-5p (both m^6^A modified) are higher in gastrointestinal cancers versus normal tissue, although the overall expressions of these miRNAs quantified by reverse transcription-polymerase chain reaction (RT-PCR) were not different between samples. When analyzing the interaction between m^5^C modified miR-200c-3p and the AGO protein it was noticed that the van der Waals interactions are stronger and the hydrogen bonding is disrupted, inducing a positional change [95].

In a more recent paper, Cheray et al. showed by using five distinct methods that a group of five miRNAs (miR-16-5p, miR-181a-5p, miR-181b-5p, miR-181d-5p, and miR-210-3p) suffer m^5^C modifications in multiple glioblastoma cell lines. Because miR-181a-5p was the most methylated in different cell lines, the authors decided to further focus on its function and discovered that the protein complex DNMT3A/AGO4 is responsible for the cytosine methylation of miRNAs in glioblastoma. Mechanistically, it was observed that the cytosine methylation of miR-181a-5p abolishes the function of this miRNA to bind the 3′UTR of the apoptotic regulator *BIM* and consequently the post-transcriptional inhibition is hindered. MiR-181a-5p is a well-known tumor suppressor miRNA in glioblastoma, capable to increase apoptosis and inhibit proliferation and invasion, but m^5^C modifications inhibits these functions, both in vitro and in vivo. Finally, these findings were also confirmed in clinical samples: high levels of unmethylated miR-181a-5p were associated with significantly longer overall survival in glioblastoma compared to methylated or low levels of the same miRNA [96].

Li et al. showed that in esophageal squamous cell carcinoma (ESCC) a lncRNA, NSUN2 methylated lncRNA (*NMR*), suffers m^5^C modifications. It was shown that *NMR* is up-regulated in ESCC and plays an important role in drug resistance and metastasis. Upstream, *NMR* transcription is activated by NF-kB and downstream *NMR* interacts with the protein BPTF, up-regulating the expression of two oncogenes: MMP3 and MMP10. Moreover, it was proven that the *NMR* suffers m^5^C modification, and the modification is promoted by the direct interaction with the writer NSUN2. The methylation of *NMR* is up-regulated in ESCC, and most probably enhances the functionality of this oncogenic lncRNA [97].

### 3.2. 5-Hydroxymethylcytosine (hm^5^C)

hm^5^C occurs especially in polyadenylated RNAs and promotes the translation of mRNAs [98]. As already mentioned, the enzymes of the TET family (TET1, TET2, TET3) that promote the oxidation of m^5^C in DNA, also are responsible for the catalyzation of m^5^C to hm^5^C in RNA in vitro [94]. TET enzymes could play a more significant role in RNA demethylation since evidence suggests that TET1 has a suppressor role in hematopoietic cancer and TET2 deletions and mutations promote tumorigenesis in myeloid leukemia and are also associated with altered DNA levels of hm^5^C [21,99,100].

Regarding ncRNAs, to-date no direct hm^5^C modifications were discovered at the RNA level in cancer. hm^5^C modifications at the RNA level are common in polyadenylated RNAs [98], hence it is reasonable to speculate that lncRNAs with poly-A tails could suffer hm^5^C modifications. Mechanistically, in mRNAs, hm^5^C modifications promote translation [98]. Multiple recent studies have reported that numerous lncRNAs contain small open reading frames (smORFs) which are translated into micropeptides [101]. Therefore, future research should be directed to further evaluate hm^5^C modifications in lncRNAs and to explore if this chemical modification affects the translation of lncRNAs.

Some indirect links between ncRNAs and hm^5^C modifications already exist. It was recently shown that, in HCC, the levels of the circRNA circTRIM33-12 are down-regulated and low levels associate with a shorter overall survival. Phenotypical, high levels of circTRIM33-12, decrease the invasiveness and the proliferation of tumor cells. Mechanistically, this circRNA, sponges miR-191 which targets TET1. Very interesting, high levels of circTRIM33-12, by derepressing TET1, increase the global level of hm^5^C and m^5^C, and plays an anti-tumorigenic role by up-regulating several tumor suppressors: TP53INP1, ULBP1 and JHDM1D [102]. Hence, future studies need to explore if circTRIM33-12/TET1 axis changes also the hm^5^C levels of ncRNAs.

At the DNA level, Hu et al. discovered that transcription of deregulated lncRNAs, in CRC, is controlled by hm^5^C modifications. The modifications control transcription of lncRNAs directly, being distributed along the lncRNA gene, or indirectly, modifying enhancer and super enhancer regions located in the proximity of the lncRNA gene. Additionally, the authors showed that certain hm^5^C DNA markers also have a practical significance, correlating with several clinical characteristics of CRC patients [103]. Therefore, we consider that it would be interesting to further study if hm^5^C modifications are present also at the lncRNA level in cancer.

### 3.3. N^6^-Methyladenosine (m^6^A)

m^6^A was first described in 1974 [104] and is the most encountered internal modification of mRNA [21,105]. The m^6^A modifications are dynamic and reversible and usually appear in the 3’ UTR region and near the stop codons [85]. These modifications are needed for the primary transcripts of miRNAs in order to develop mature miRNAs [106]. The methylation of adenosine is facilitated by members of the methyltransferase like (METTL) proteins family, which act as writers [85]. METTL3 is the core of the catalysis process, while METTL14 acts as a support protein and Wilms’ tumor 1-assoaciating protein (WTAP) identifies the METTL3-METTL14 complex [85,107,108]. Cui et al. demonstrated that, by knocking-down METTL3 or METTL14, the growth and self-renewal of glioblastoma stem cells are promoted [109]. High METTL3 levels are associated with worse prognosis in HCC patients [110]. Pendleton et al. recently showed that METTL16 can function independently of METTL3 [111] and has a role in the maturation of metastasis associated lung adenocarcinoma transcript 1 (*MALAT1*) lncRNA, which executes a tumor suppressor or promoting role, depending on the type of cancer involved [112]. Additionally, Brown et al. demonstrated that the METTL16 binds at the 3′ UTR triple helical region of *MALAT1* [113]. Moreover, m^6^A changes in *MALAT1* alter its binding capacity of miRNAs involved in cancer [114]. RNA-binding motif protein 15 (RBM15) and RMB15B are also part of the METTL3 complex and target the X-inactive specific transcript (*XIST*) lncRNA [115]. *XIST* has a pro tumorigenic role in multiple types of cancer such as gastric, colorectal, and pancreatic [84]. MiRNAs can influence the formation of m^6^A by altering the binding of METTL3 to mRNAs that contain miRNA targeting sites [116].

m^6^A binding proteins (readers) act as mediators for the majority of the roles of m^6^A writer and eraser enzymes and this makes them a target for therapy due to their involvement in cancer [83]. The YT521-B homology domain family (YTHDF) proteins are readers that are preponderantly cytoplasmic (YTHDF1, YTHDF2, YTHDF3), nuclear (YTHDC1), or nuclear cytoplasmic (YTHDC2) [117]. Bai et al. demonstrated that high levels of YTHDF1 promote tumorigenesis in CRC [118]. YTHDF2 alters the stability of mRNAs and ncRNAs containing m^6^A [119] and has a role in the oncogenesis of acute myeloid leukemia (AML), where its’ overexpression prevents the apoptosis of leukemic stem cells by down-regulating the tumor necrosis factor receptor 2 (TNFR2) [120]. YTHDC1 mediates the function of *XIST* by binding to its’ m^6^A residues [115]. Tanabe et al. showed that YTHDC2 is overexpressed in CRC and promotes the metastasis by targeting HIF1A [121]. HNRNPA2B1 facilitates the formation of mature miRNAs, by m^6^A binding in some subsets of primary miRNA transcripts and subsequently interacting with DGCR8 protein [122]. Besides this, reducing m^6^A levels prevents the maturation of miRNAs, such as the tumor suppressor let-7 [21,122].

The two m^6^A erasers, fat mass and obesity associated protein (FTO) and 𝛼-ketoglutarate-dependent dioxygenase alkB homolog 5 (ALKBH5), belong to the Fe(II) and 𝛼-KG-dependent dioxygenase AlkB family [123,124]. FTO promotes leukemogenesis in AML by down-regulating the m^6^A levels of Ankyrin Repeat and SOCS Box Containing 2 (ASB2) and Retinoic Acid Receptor Alpha (RARA) [125]. Also, FTO has an oncogenic role and decreases the immunotherapeutic response in melanoma, by m^6^A demethylation of specific mRNAs such as Programmed cell death protein 1 (PD1), *CXC* motif Chemokine Receptor 4 (CXCR4) and SRY-Box Transcription Factor *1* (SOX1) [126]. By treating glioblastoma stem cells with the FTO inhibitor MA2 the tumor progression can be suppressed [109]. ALKBH5 is also involved in the progression of glioma and its overexpression is associated with a poor prognosis [127]. ALKBH5 demethylates Forkhead Box M1 (FOXM1) transcripts and consequently increased the levels of FOXM1. The interaction between the ALKBH5 and nascent FOXM1 transcripts is mediated by *FOXM1-AS*, a lncRNA. Therefore, by depleting ALKHB5 and *FOXM1-AS*, the oncogenic process of glioblastoma stem cells can be altered [127]. ALKBH5 overexpression induced by HIF1A reduces the methylation of *NANOG* mRNA, leading to increased expression of NANOG and breast cancer stem cells [128]. By knocking-down ALKBH5 the oncogenesis in breast cancer can be altered through a lower number of BCSC [128].

In gastrointestinal cancers (pancreatic cancer and CRC tissues), let-7a-5p and miR-17-5p show enhanced m^6^A modifications compared to normal tissue samples. By studying the interaction between the AGO2 protein with let-7a-5p and miR-17-5p, in their methylated and non-methylated forms, Konno et al. observed that m^6^A modifications lead to conformational changes in the complexes structure and a difference in the space size of the RNA recognition site, even though m^6^As are not in the proximity of the RNA-binding site. Consequently, target mRNAs translation is not suppressed anymore [95].

### 3.4. N^1^-Methyladenosine (m^1^A)

The m^1^A modification has been described in tRNAs [129], but recent data show that it is also present in mRNAs, in the 5′-UTR [130,131]. Zhou et al. demonstrated that in a small subtype of cytosolic mRNAs, m^1^A was most abundant in the 3′-UTR and the coding sequence [132], therefore m^1^A modifications might exist at a well-defined stoichiometry in only a subtype of cytosolic mRNAs [83].

Dai et al. showed that members of the YTH protein family (YTHDF1, YTHDF2, YTHDF3 and YTHDC1) that are readers for m^6^A, could also bind to m^1^A, but without the nuclear cytoplasmic YTHCD2 protein [133]. The writers for m^1^A in tRNAs are tRNA methyltransferase 10 homologue A (TRM10) and the complex formed *by* tRNA methyltransferase non-catalytic subunit 6 (TRM6) and tRNA methyltransferase catalytic subunit 61 (TRM61) [129]. For mRNA, the tRNA-like motif GUUCRA is identified by the TRM6/61A complex, as for lncRNAs there are no specific writers described, but the presence of the GUUCRA motif can undergo m^1^A methylation. For example, *MALAT1* lncRNA, typically overexpressed in cancers [134], is m^1^A methylated in the A8398 position [135,136,137]. Two erasers can revert the m^1^A modification, ALKBH1 and ALKBH3 [130,138]. ALKBH3 can enhance the proliferation, migration and invasion of cancer cells by demethylating tRNAs [139]. Particularly, ALKBH3 can promote the invasion of breast and ovarian cancer cells by m^1^A demethylation of colony stimulating factor 1 (*CSF-1*) mRNA [140]. Also, overexpression of ALKBH3 is known to promote the proliferation and angiogenesis in pancreatic cancer [141].

### 3.5. Pseudouridylation (Ψ)

Pseudouridylation (5-ribosyluracil) (Ψ) was first described in 1951 and is the most encountered modification in ncRNAs [142,143] and it is present in lncRNAs (i.e., *MALAT1*, *XIST*), tRNAs, rRNAs and small nucleolar RNAs (snoRNAs) [21]. Ψ is formed through the C-N to C-C isomerization of uridine that forms an additional hydrogen bond [144]. Ψ binds to adenosine in similar way as uridine, but forms a much stronger bond with the other bases [85]. The writers for Ψ are pseudouridine synthases (PUSs), which can be RNA dependent or RNA-independent. An additional adjuvant writer is dyskerin pseudouridine synthase 1 (DKC1) which mainly targets ncRNAs [145]. Up-regulation of DKC1 is associated with worse prognosis in lung and pancreatic cancer [146,147]. Moreover, altering DCK1 can lead to inactivation of tumor suppressors, such as p53 which was seen in breast cancer [148,149]. PUS1 plays a role in the interaction between steroid receptor RNA activator 1 (SRA1) with the retinoic acid receptor-gamma (RARG) and the estrogen receptor in melanoma and breast cancer cells, respectively [150]. Depletion of PUS10 prevents the apoptosis of p53-null prostate cancer cells determined by the TNF-related apoptosis inducing ligand (TRAIL) [151].

Regarding ncRNAs, Ψ has been described in certain ncRNAs [152,153], which in other studies were reported to be dysregulated in multiple cancers. However, there is no clear evidence that the presence of Ψ in these ncRNAs also impacts their functions or expressions in cancer. For example, the lncRNA zinc finger antisense 1 (*ZFAS1*) which has been reported to contain a pseudouridine molecule [84,153], is dysregulated in several types of cancer–ranging from bladder [154] to lung, colon, liver, and gastric cancer [155].

Interestingly, the telomerase RNA component (*TERC*) is also a pseudouridylated lncRNA, known to be implicated in modulating the telomere length and was linked to cellular aging and chromosomal instability [156]. On the other hand, higher levels of *TERC* and consequentially increased telomerase activity were reported in lung cancer cells [146]. Likewise, consistent overexpression of TERC has been reported in prostate cancer, and MYC mediated down-regulation of *TERC* has been achieved in several cancer cell lines, speculating its potential therapeutic use [157].

Small nucleolar RNA host gene 1 and 7 (*SNHG1*, *SNHG7*) are two pseudouridylated lncRNAs [152,158] with oncologic implications. While *SNHG7* expression interference has been shown to affect gastric cancer development and progression both in vitro and in vivo [159], *SHNG1* has been shown to promote CRC proliferation by sponging miR-145, a known tumor suppressor [160]. Likewise, it has been demonstrated that *SNHG1* might also be implicated in glioma tumorigenesis and progression by sponging miR-194 and preventing it from binding to its downstream targets [161]. However, the direct effects of Ψ on these ncRNA has not yet been demonstrated and needs to be further elucidated.

### 3.6. Uridylation

Uridylation represents the addition of one or more uridine molecules to the 3′ end of various transcripts, which include mRNA and ncRNAs, including their precursor molecules [83]. A reported writer is the terminal uridylyltransferase (TUT1) enzyme which by uridylation is responsible for the physiological maturation of the U6 small nuclear RNA (snRNA) of the spliceosome [83,162].

In osteosarcoma, TUT1, which has been found to be down-regulated, has been reported to affect lipogenesis (via miR-24 and miR-29 and their targets PPARgamma and SREBP-1c) and therefore acts as a tumor suppressor [163]. Most probably TUT1 regulates the levels of miR-24 and miR-29 by 3′ nucleotide additions of uridine molecules. Likewise, the TUT1 down-regulation in breast cancer negatively influences the stability of the pro-apoptotic factor BCL-2-interacting-killer (BIK), also acting as a tumor suppressor [164]. Other writers, the TUT4 and TUT7, are known to be implicated in the maturation process of let-7 family. A LIN28 mediated blockage in maturation of pre-let-7 ncRNA was linked to head and neck cancer [165], as well as breast cancer [166], most probably the mechanism is mediated also via TUT4 and TUT7 which play oncogenic roles [167,168]. DIS3-like exonuclease 2 (DIS3L2) has been reported as a potential reader, its mutation being linked to the Perlman syndrome and Wilms tumor. However, it is not clear if polyuridylation is the direct responsible for the pro-oncogenic effect of this reader [83,169,170].

### 3.7. 7-Methylguanosine (m^7^G)

The internal methylation of the guanosine molecule at its seventh position is a known modification in rRNAs and tRNAs, with recent reports of this modification occurring internally also in mRNAs, miRNAs and miRNA precursors [171].

This modification is essential in pri-miRNA processing by physically preventing guanosine quadruplex formation (Hoogsteen base pairing) [83]. Methyltransferase like 1 (METTL1) enzyme has been reported as a potential writer in lung cancer and colon cancer cells. It is implicated in the normal maturation of let-7e precursors, consequentially, its down-regulation increasing the expression of its target gene *HMGA2,* implicated in the tumor migration potential [171]. Moreover, in other cancers, like glioblastoma and HCC, the high expression levels of METTL1 have been linked to a poorer prognosis, by unknown mechanisms. While high METTL1 levels might be implicated in the cancer cell homeostasis by stabilizing tRNA, its inactivation decreases the pseudourydilation and the 7-guanyl-methylation, which in turn destabilize the tRNAs [83,172,173]. Additionally, it is well known that an overexpression of mRNA cap guanine-N7 methyltransferase (RNMT), can promote tumorigenesis in mammary epithelial cells [174], but this kind of modification is not yet described in ncRNAs.

In Figure 2, we show the chemical structure of ncRNA modifications, their writers, erasers, and readers that were recently described, and in Table 2, we present an illustrative list of ncRNA chemical modifications and their impact on oncogenesis.

## 4. Future Perspectives

The functions of many ncRNAs remain poorly characterized and a detailed mechanistic description is missing. Often the sole argument supporting the functional roles for ncRNAs in cancer pathogenesis stems from expression studies. The fact that a given ncRNAs is overexpressed in cancer makes it an oncogene, and on the other hand, a down-regulated ncRNA in cancer is a priori considered a tumor suppressor gene. This type of research is often misleading, considering possible by products of the malignant disease to be oncogenes and ignoring molecules that do not change their expressions in cancer. We consider that ncRNAs that do not change their expressions between healthy status and cancer could play important pathogenic roles in the malignant disease, could be potential therapeutic targets, could be addictive genes, and essential regulators of the cancer cell fate.

For example, we observed in different physiological/pathological situations very high correlations between the expressions of miRNAs from different patient samples and we translated these correlations into miRNA networks [175,176,177,178]. Initially, we discovered in sepsis that the correlations between circulating plasma miRNAs disappear, the miRNA network is fragmented, and many miRNA nodes are isolated in comparison to the miRNA network of healthy controls. One possible explanation for these changes could be the editing and chemical modifications of specific miRNAs in sepsis [175]. In a subsequent paper, we observed that at the time of diagnosis of chronic lymphocytic leukemia (CLL), the expressions of miRNAs do not correlate very well (i.e., the miRNA network is not well connected) and the miRNA expressions in samples from the same patients, after Richter transformation, show a very high level of correlations, although most of the miRNAs were not up-/down-regulated compared to CLL at diagnosis (i.e., the miRNA network is very well connected). Oppositely, the miRNA network of patients with CLL after a long fallow-up (but which do not develop Richter syndrome) is identical to the miRNA network at the diagnosis of CLL [177]. A similar phenomenon was observed also by Volinia et al. who described a reorganization of the miRNA expression network in multiple types of solid and hematological cancers [179].

Very interesting, the most connected miRNAs in the network (hubs) are often the ones which are neither up- nor down-regulated between healthy controls versus cancer/sepsis patients. We consider that this phenomenon can be explained only by changes that individually and specifically affect each miRNA type, similarly to the ones induced by editing or chemical modifications. In other words, we insinuate that the appearance/disappearance of an edge (i.e., correlation), between two miRNAs, in a correlation-based miRNA network could reflect changes in miRNA editing or chemical modifications. Understanding these concepts and discovering the precise mechanisms of ncRNA editing and chemical modifications can open a new era of biomarker and therapeutic studies.

In the past 20 years, hundreds of studies tried to establish ncRNAs as the next generation of circulating cancer biomarkers. This concept is very appealing as ncRNAs circulating in bodily fluids seem to be ideal non-invasive biomarkers. One of the main problems that blocked the translation of this data from bench to bedside is the lack of specificity of ncRNAs in diagnosing a particular type of cancer. The same up-/down-regulated miRNAs, lncRNAs or circRNAs were proposed as non-invasive biomarkers for multiple types of cancer. Hence, an alternative approach is necessary, such that the focus will be less on quantitative changes and more on qualitative changes. The best diagnostic ncRNA will not be the one deregulated in bodily fluids of cancer patients, but the one both deregulated and edited/chemically modified. The latter being the element that will offer the missing specificity to ncRNA as biomarkers. Elegantly, Konno et al., showed that the methylation level of miR-17-5p in serum from early pancreatic cancer patients is significantly higher compared to healthy controls. Moreover, the authors prove that methylated miR-17-5p is more specific than carbohydrate antigen 19-9 (CA19-9) and carcinoembryonic antigen (CAE) [95]. Additional large studies are highly necessary to further support the diagnostic power of edited and chemically modified ncRNAs in cancer.

Moreover, the enzymes that are responsible for the editing and modification could also be of value, as their expressions are changed in cancerous tissue compared to normal tissue. APOBEC3B level is a prognosis marker that correlates well with poor outcomes in positive estrogen-receptor breast cancers [180]; ADAR1 over-expression is correlated with higher TNM stages in gastric cancer patients [181,182]; and the examples could continue. m6A regulators are also potential biomarkers: up-regulation of METTL3 is correlated with an adverse prognosis in HCC [183]; FTO is up-regulated in breast cancer and its high levels are associated with lower survival rates [184]; YTHDF3 is a significant prognostic factor for poor overall survival in CRC patients [185].

In the last years, we have witnessed the development of ncRNA therapeutics [186,187,188]. NcRNA therapy in cancer is characterized by targeting up-regulated oncogenic or restoring down-regulated tumor suppressor ncRNAs. Again, we underline that target selection is based mainly on quantitative criteria and less on qualitative criteria. By studying chemically modified or edited ncRNAs new specific targets can be selected. For example, in metastatic melanoma miR-378a-3p does not suffer any more A-to-I editing and its target, the oncogene *PARVA*, is not suppressed any more, and promotes invasion and metastasis [44]. This type of mechanistic study unveils a new therapeutic strategy for melanoma, which could inhibit metastasis. By restoring the expression of edited miRNA, using miRNA mimetics and/or by activating ADAR1, the enzyme responsible for the editing, melanoma progression might be inhibited. Furthermore, by inducing chemical modifications in small interfering RNAs (siRNAs) (such as sugar or phosphate backbone modifications), their in vivo stability could be improved, and therefore, their therapeutic effects could be increased in cancer [189].

RNA editings and RNA modifications offer a novel layer of complexity to the intricate mechanisms of cancer development and therapeutic resistance. The heterogeneity of cancer cells, clonal selection and repopulation are mechanisms of cancer relapse, but determining the exact profile of each subpopulation, permits personalized treatment. It is like putting more detail to a canvas and ultimately recognizing the characters in it.

Pharmacological modulation of writers, readers and erasers by inhibitors or activators, has a huge therapeutic potential. Looking at the m^6^A modification of ncRNAs, we can realize that the field of RNA-modifying proteins as drug targets is constantly expanding [190]. Inhibition of METTL3 by using intratumoral siRNA decreased the tumor growth rate and tumor weights in two xenograft models of CRC [191]. Substrate competitive inhibitors of METTL3 have also been identified and structurally characterized [192], but there are still the problems of their functionality and selectivity in vivo, as well as delivery to the targeted cells. Furthermore, these small molecules may serve to the design of inhibitors based on protein-protein interaction. Moreover, the development of proteolysis-targeting chimeras (PROTACs) that induce targeted protein degradation by the ubiquitin–proteasome system [193], may be a way of modulating the oncogenic epitranscriptomic regulators. Inhibitors of m^6^A erasers, like FTO, are also of great interest. For example, meclofenamic acid enhances the effect of temozolomide on suppressing proliferation of glioma cells [194] and suppresses tumor progression of glioblastoma stem cells [109]. Animal xenograft studies demonstrated a potent therapeutic efficacy of CS1 (bisantrene) and CS2 (brequinar) in treating AML, through inhibition of FTO. CS1 and CS2 sensitize AML cells to T-cell cytotoxicity, thus overcoming immune evasion [195]. Similarly, FB23-2, another FTO inhibitor, can significantly decrease progression of AML cell lines and primary cells in xeno-transplanted mice [196].

## 5. Conclusions

Deregulated ncRNAs expression is a fundamental characteristic of cancer cells and of the malignant microenvironment. Unfortunately, this intrinsic characteristic is not yet exploited in the clinical setting. NcRNAs are not only post-transcriptional regulators, but are themselves regulated at the RNA level, being edited and chemically modified. We believe that a better understanding of the mechanisms that regulate the function of ncRNAs could be the breakthrough that will implement these molecules as future diagnostic and therapeutic tools.

## Figures and Tables

**Table 1:** An illustrative list of ncRNAs aberrantly edited in cancer.

Cancer	ncRNA	Enzyme	Editing	Effects	Consequence	Ref
Breast, lung, ovarian, renal cancer cell lines	miR-379-5p	ADAR2	↓	↑ ADGRE5	cell proliferation and inhibited apoptosis	[36]
Breast, ovarian, renal cancer cell lines	mir-200b-3p	ADAR1/2	↑	↑ ZEB1/2 ↓ LIFR	cell invasion and migration	[34]
Glioblastoma	miR-589-3p	ADAR2	↓	↓ PCDH2 ↑ ADAM12	cell proliferation, invasion and migration	[42]
Glioblastoma	miR-221/222 miR-21	ADAR2	↓	↓ p27Kip1	cell proliferation	[40]
Glioblastoma	miR-376a cluster	ADAR1	↓	↓ RAP2A ↑ AMFR	cell invasion and migration	[41]
Hepatocellular carcinoma	miR-214	ADAR2	↑	↑ RAB15	cell proliferation, invasion, angiogenesis	[45]
Leukemia	let-7	ADAR1	↑	↑ LIN28B	enhanced self-renewal	[46]
Melanoma	miR-378a-3p	ADAR1	↓	↑ PARVA	metastasis	[44]
Melanoma	miR-455-5p	ADAR1	↓	↓ CPEB1	tumor growth and metastasis	[43]
NSCLC	miR-381-3p	ADAR1	↑	N/A	cell proliferation, invasion	[47]
MDS/ MPN	*CCAT2* at the rs6983267 SNP	N/A	↑	Homozygous GG/TT -> heterozygous G/T	low risk MDS	[29]

ADAM12 = disintegrin and metalloproteinase domain-containing protein 12; ADGRE5 = adhesion G protein–coupled receptor E5; AMFR = autocrine motility factor receptor; CPEB1 = cytoplasmic polyadenylation element binding protein 1, ID-1 = inhibitor of differentiation 1; p27Kip1 = cyclin-dependent kinase inhibitor 1B; LIFR = leukemia inhibitory factor receptor; MDS = myelodysplastic syndrome; MPN = myeloproliferative neoplasms; N/A = not available; NSCLC = non-small-cell lung cancer; PCDH9 = protocadherin 9; RAP2A = member of the RAS oncogene family; RAB15 = member RAS oncogene family; SNP = single nucleotide polymorphism; ZEB1/2 = Zinc finger E-box-binding homeobox 1/2, ↓ = decreased; ↑ = increased.

**Table 2:** An illustrative list of ncRNAs aberrantly chemically modified in cancer.

Chem. Mod.	Cancer	ncRNA	Enzyme	Mod	Effects	Consequence	Ref
m^5^C	Pancreatic cancer and colorectal cancer	miR-200c-3p, miR-21-3p	NSUN2	↑	interaction between miRNA and AGO is modified	N/A	[95]
	GBM	miR-16-5p, miR-181a-5p, miR-181b-5p, miR-181d-5p, miR-210-3p	DNMT3A/ AGO4	↑	Cytosine methylated miR-181a-5p loses its capacity to suppress BIM (apoptosis regulator)	Decreases apoptosis and increases invasion and proliferation rate.	[96]
	ESCC	*NMR*	NSUN2	↑	Attenuates the methylation of PLOD3, COL4A5, LAMB1, HSPG2	Increases migration and invasion	[97]
m^6^A	Pancreatic cancer and colorectal cancer	let-7a-5p, miR-17-5p	METTL3 and METTL4	↑	interaction between miRNA and AGO is modified	m^6^A modified miRNAs have a reduced ability to inhibit mRNAs	[95]
Ψ	Lung cancer	*TERC*	N/A	N/A	N/A	Telomere shortening, pro-oncogenic	[146]
Uridylation	Osteosarcoma	miR-24, miR-29a	TUT1	N/A	↑ PPARgamma ↑ SREBP-1c	Stimulates lipogenesis, tumor progression	[163]
	Breast cancer	let-7a, let-7f	LIN28	↑	↑ HRAS, ↑ HMGA2,	Expansion of cancer stem cells	[166]
	Head and Neck cancer	let-7 family	LIN28B	↑	↑ HMGA2, ↑ CCND2, ↑ IGF1R, ↑ IGF2BP2	Oncogenesis and cancer progression	[165]
m^7^G	Lung and colon cancer cells	let-7 family (let-7-5p seed), hsa-miR-125a-5p, hsa-miR-92b-3p	METTL1	↑	↓ HMGA2	Cell migration	[171]

AGO = Argonaute; AGO4 = Argonaute RISC catalytic component 4; BIM = BCL2 like 11; CCND2 = cyclin D2; COL4A5 = collagen type IV alpha 5 chain; DNMT3A = DNA (cytosine-5)-methyltransferase 3A; ESCC = esophageal squamous cell carcinoma; GBM = Glioblastoma multiforme; HMGA2 = High mobility group protein HMGI-C; HRAS = GTPase HRas; HSPG2 = heparan sulfate proteoglycan 2; IGF1R = insulin like growth factor 1 receptor; IGF2BP2 = insulin like growth factor 2 mRNA binding protein 2; LIN28B = lin-28 homolog B; LAMB1 = laminin subunit beta 1; m^5^C = 5-methylcytosine; m^6^A= N^6^-Methyladenosine; m^7^G = 7-Methylguanosine; METTL1 = (guanine-N(7)-)-methyltransferase 1; METTL3 = N6-adenosine-methyltransferase catalytic subunit; METTL4 = N(6)-adenine-specific methyltransferase; NSUN2 = Myc-induced SUN domain-containing protein; PLOD3 = procollagen-lysine,2-oxoglutarate 5-dioxygenase 3; PPARgamma = peroxisome proliferator-activated receptor gamma; Ψ = pseudouridylation; SREBP-1c = Sterol regulatory element-binding protein 1; TUT1 = terminal uridylyl transferase 1; TUT4 = terminal uridylyltransferase 4; TUT7 = terminal uridylyltransferase 7, ↓ = decreased; ↑ = increased.

## Data Availability

Not applicable.

## Abbreviations

A-to-I	adenosine to inosine
ACF	Apobec-1 complementation factor
ADAR	adenosine deaminase acting on RNA
ALKBH5	alpha-ketoglutarate-dependent dioxygenase alkB homolog 5
AMFR	autocrine motility factor receptor
AML	acute myeloid leukemia
APOBEC	apolipoprotein B mRNA editing enzyme, catalytic polypeptide-like
ASB2	ankyrin repeat and SOCS box containing 2
BIK	BCL-2-interacting-killer
C-to-U	cytidine to uridine
CA19-9	carbohydrate antigen 19-9
CAE	carcinoembryonic antigen
cDNA	complementary DNA
circRNA	circular RNA
CLL	chronic lymphocytic leukemia
CSF-1	colony stimulating factor 1
CXCR4	CXC motif chemokine receptor 4
DIS3L2	DIS3-like exonuclease 2
DKC1	dyskerin pseudouridine synthase 1
DND1	dead-end protein homolog 1
DNMT	DNA methyltransferases
DRAI	DNA-to-RNA allelic imbalance
ESCC	esophageal squamous cell carcinoma
FOXM1	forkhead box M1
FTO	fat mass and obesity associated protein
GAC	glutaminase isoform C
gDNA	genomic DNA
GLS	glutaminase
hm5C	5-hydroxymethylcytosine
KGA	glutaminase kidney isoform
LIFR	leukemia inhibitory factor receptor
lncRNA	long non-coding RNA
LTR	long terminal repeat
m1A	N1-Methyladenosine
m5C	5-methylcytosine
m6A	N6-Methyladenosine
MALAT1 MALDI-TOF-MS	metastasis associated lung adenocarcinoma transcript 1 matrix-assisted laser desorption/ionization-time of flight- mass spectrometry
MDS	myelodysplastic syndrome
METTL1	methyltransferase like 1
miRNA	microRNA
MMP2	matrix metallopeptidase 2
MPN	myeloproliferative neoplasm
ncRNA	non-coding RNA
NMR	NSUN2 methylated lncRNA
NSCLC	non-small-cell lung cancer
NSUN	NOP2/Sun RNA methyltransferase
PARVA	alpha-parvin
PD1	programmed cell death protein 1
pre-miRNA	precursor miRNA
pri-miRNA	primary miRNA
PROTAC	proteolysis-targeting chimera
PUS	pseudouridine synthase
RARA	retinoic acid receptor alpha
RARG	retinoic acid receptor gamma
RBM15	RNA-binding motif protein 15
RDD	RNA-DNA difference
rRNA RT-PCR	ribosomal RNA reverse transcription-polymerase chain reaction
siRNA	small interfering RNA
SNHG1	Small nucleolar RNA host gene 1
SNHG7 snoRNA	Small nucleolar RNA host gene 7 small nucleolar RNA
SNP snRNA	single nucleotide polymorphism small nuclear RNA
SOX1	SRY-Box transcription factor 1
SRA1	steroid receptor RNA activator 1
TERC	telomerase RNA component
TETt	en-eleven family demethylases
TNFR2	tumor necrosis factor receptor 2
TRAIL	TNF-related apoptosis inducing ligand
TRM10	tRNA methyltransferase 10 homologue A
TRM6	tRNA methyltransferase non-catalytic subunit 6
TRM61	tRNA methyltransferase catalytic subunit 61
tRNA	transfer RNA
TUT1	terminal uridylyltransferase 1
XIST	X-inactive specific transcript
ZFAS1	zinc finger antisense 1
Ψ	pseudouridylation

## References

[1] Venter J.C., Adams M.D., Myers E.W., Li P.W., Mural R.J., Sutton G.G., Smith H.O., Yandell M., Evans C.A., Holt R.A. (2001). The sequence of the human genome. Science.

[2] Kapranov P., Cheng J., Dike S., Nix D.A., Duttagupta R., Willingham A.T., Stadler P.F., Hertel J., Hackermuller J., Hofacker I.L. (2007). RNA maps reveal new RNA classes and a possible function for pervasive transcription. Science.

[3] Dragomir M.P., Kopetz S., Ajani J.A., Calin G.A. (2020). Non-coding RNAs in GI cancers: From cancer hallmarks to clinical utility. Gut.

[4] Silva A., Bullock M., Calin G. (2015). The Clinical Relevance of Long Non-Coding RNAs in Cancer. Cancers.

[5] Fabbri M., Calore F., Paone A., Galli R., Calin G.A. (2013). Epigenetic regulation of miRNAs in cancer. Adv. Exp. Med. Biol..

[6] Bartel D.P. (2004). MicroRNAs: Genomics, biogenesis, mechanism, and function. Cell.

[7] Michlewski G., Caceres J.F. (2019). Post-transcriptional control of miRNA biogenesis. RNA.

[8] Hausser J., Zavolan M. (2014). Identification and consequences of miRNA-target interactions—Beyond repression of gene expression. Nat. Rev. Genet..

[9] Ponting C.P., Oliver P.L., Reik W. (2009). Evolution and functions of long noncoding RNAs. Cell.

[10] Kopp F., Mendell J.T. (2018). Functional Classification and Experimental Dissection of Long Noncoding RNAs. Cell.

[11] Yu C.Y., Kuo H.C. (2019). The emerging roles and functions of circular RNAs and their generation. J. Biomed. Sci..

[12] Calin G.A., Dumitru C.D., Shimizu M., Bichi R., Zupo S., Noch E., Aldler H., Rattan S., Keating M., Rai K. (2002). Frequent deletions and down-regulation of micro- RNA genes miR15 and miR16 at 13q14 in chronic lymphocytic leukemia. Proc. Natl. Acad. Sci. USA.

[13] Nicoloso M.S., Spizzo R., Shimizu M., Rossi S., Calin G.A. (2009). MicroRNAs—The micro steering wheel of tumour metastases. Nat. Rev. Cancer.

[14] Spizzo R., Nicoloso M.S., Croce C.M., Calin G.A. (2009). SnapShot: MicroRNAs in Cancer. Cell.

[15] Schmitt A.M., Chang H.Y. (2016). Long Noncoding RNAs in Cancer Pathways. Cancer Cell.

[16] Kristensen L.S., Hansen T.B., Veno M.T., Kjems J. (2018). Circular RNAs in cancer: Opportunities and challenges in the field. Oncogene.

[17] Dragomir M., Calin G.A. (2018). Circular RNAs in Cancer—Lessons Learned From microRNAs. Front. Oncol.

[18] Anfossi S., Babayan A., Pantel K., Calin G.A. (2018). Clinical utility of circulating non-coding RNAs—An update. Nat. Rev. Clin. Oncol..

[19] Pardini B., Sabo A.A., Birolo G., Calin G.A. (2019). Noncoding RNAs in Extracellular Fluids as Cancer Biomarkers: The New Frontier of Liquid Biopsies. Cancers.

[20] Morales S., Monzo M., Navarro A. (2017). Epigenetic regulation mechanisms of microRNA expression. Biomol. Concepts.

[21] Esteller M., Pandolfi P.P. (2017). The Epitranscriptome of Noncoding RNAs in Cancer. Cancer Discov..

[22] Squires J.E., Patel H.R., Nousch M., Sibbritt T., Humphreys D.T., Parker B.J., Suter C.M., Preiss T. (2012). Widespread occurrence of 5-methylcytosine in human coding and non-coding RNA. Nucleic Acids Res..

[23] Christofi T., Zaravinos A. (2019). RNA editing in the forefront of epitranscriptomics and human health. J. Transl. Med..

[24] Picardi E., D’Erchia A.M., Gallo A., Montalvo A., Pesole G. (2014). Uncovering RNA Editing Sites in Long Non-Coding RNAs. Front. Bioeng. Biotechnol..

[25] Zheng Y., Ji B., Song R., Wang S., Li T., Zhang X., Chen K., Li T., Li J. (2016). Accurate detection for a wide range of mutation and editing sites of microRNAs from small RNA high-throughput sequencing profiles. Nucleic Acids Res..

[26] Ramaswami G., Li J.B. (2016). Identification of human RNA editing sites: A historical perspective. Methods.

[27] Distefano R., Nigita G., Veneziano D., Romano G., Croce C.M., Acunzo M. (2019). isoTar: Consensus Target Prediction with Enrichment Analysis for MicroRNAs Harboring Editing Sites and Other Variations. Methods Mol. Biol..

[28] Nigita G., Marceca G.P., Tomasello L., Distefano R., Calore F., Veneziano D., Romano G., Nana-Sinkam S.P., Acunzo M., Croce C.M. (2019). ncRNA Editing: Functional Characterization and Computational Resources. Methods Mol. Biol..

[29] Shah M.Y., Ferracin M., Pileczki V., Chen B., Redis R., Fabris L., Zhang X., Ivan C., Shimizu M., Rodriguez-Aguayo C. (2018). Cancer-associated rs6983267 SNP and its accompanying long noncoding RNA CCAT2 induce myeloid malignancies via unique SNP-specific RNA mutations. Genome Res..

[30] Zinshteyn B., Nishikura K. (2009). Adenosine-to-inosine RNA editing. Wiley Interdiscip. Rev. Syst Biol. Med..

[31] Walkley C.R., Li J.B. (2017). Rewriting the transcriptome: Adenosine-to-inosine RNA editing by ADARs. Genome Biol..

[32] Heale B.S., Keegan L.P., O’Connell M.A. (2010). The effect of RNA editing and ADARs on miRNA biogenesis and function. Adv. Exp. Med. Biol..

[33] Kawahara Y., Megraw M., Kreider E., Iizasa H., Valente L., Hatzigeorgiou A.G., Nishikura K. (2008). Frequency and fate of microRNA editing in human brain. Nucleic Acids Res..

[34] Wang Y., Xu X., Yu S., Jeong K.J., Zhou Z., Han L., Tsang Y.H., Li J., Chen H., Mangala L.S. (2017). Systematic characterization of A-to-I RNA editing hotspots in microRNAs across human cancers. Genome Res..

[35] Pinto Y., Buchumenski I., Levanon E.Y., Eisenberg E. (2018). Human cancer tissues exhibit reduced A-to-I editing of miRNAs coupled with elevated editing of their targets. Nucleic Acids Res..

[36] Xu X., Wang Y., Mojumdar K., Zhou Z., Jeong K.J., Mangala L.S., Yu S., Tsang Y.H., Rodriguez-Aguayo C., Lu Y. (2019). A-to-I-edited miRNA-379-5p inhibits cancer cell proliferation through CD97-induced apoptosis. J. Clin. Invest..

[37] Vaikari V.P., Yang J., Wu S., Alachkar H. (2019). CD97 expression is associated with poor overall survival in acute myeloid leukemia. Exp. Hematol..

[38] He Z., Wu H., Jiao Y., Zheng J. (2015). Expression and prognostic value of CD97 and its ligand CD55 in pancreatic cancer. Oncol. Lett..

[39] Liu D., Li C., Trojanowicz B., Li X., Shi D., Zhan C., Wang Z., Chen L. (2016). CD97 promotion of gastric carcinoma lymphatic metastasis is exosome dependent. Gastric Cancer.

[40] Tomaselli S., Galeano F., Alon S., Raho S., Galardi S., Polito V.A., Presutti C., Vincenti S., Eisenberg E., Locatelli F. (2015). Modulation of microRNA editing, expression and processing by ADAR2 deaminase in glioblastoma. Genome Biol..

[41] Choudhury Y., Tay F.C., Lam D.H., Sandanaraj E., Tang C., Ang B.T., Wang S. (2012). Attenuated adenosine-to-inosine editing of microRNA-376a* promotes invasiveness of glioblastoma cells. J. Clin. Investig..

[42] Cesarini V., Silvestris D.A., Tassinari V., Tomaselli S., Alon S., Eisenberg E., Locatelli F., Gallo A. (2018). ADAR2/miR-589-3p axis controls glioblastoma cell migration/invasion. Nucleic Acids Res..

[43] Shoshan E., Mobley A.K., Braeuer R.R., Kamiya T., Huang L., Vasquez M.E., Salameh A., Lee H.J., Kim S.J., Ivan C. (2015). Reduced adenosine-to-inosine miR-455-5p editing promotes melanoma growth and metastasis. Nat. Cell Biol..

[44] Velazquez-Torres G., Shoshan E., Ivan C., Huang L., Fuentes-Mattei E., Paret H., Kim S.J., Rodriguez-Aguayo C., Xie V., Brooks D. (2018). A-to-I miR-378a-3p editing can prevent melanoma progression via regulation of PARVA expression. Nat. Commun..

[45] Liu W.H., Chen C.H., Yeh K.H., Li C.L., Wu Y.J., Chen D.S., Chen P.J., Yeh S.H. (2013). ADAR2-mediated editing of miR-214 and miR-122 precursor and antisense RNA transcripts in liver cancers. PLoS ONE.

[46] Zipeto M.A., Court A.C., Sadarangani A., Delos Santos N.P., Balaian L., Chun H.J., Pineda G., Morris S.R., Mason C.N., Geron I. (2016). ADAR1 Activation Drives Leukemia Stem Cell Self-Renewal by Impairing Let-7 Biogenesis. Cell Stem Cell.

[47] Anadon C., Guil S., Simo-Riudalbas L., Moutinho C., Setien F., Martinez-Cardus A., Moran S., Villanueva A., Calaf M., Vidal A. (2016). Gene amplification-associated overexpression of the RNA editing enzyme ADAR1 enhances human lung tumorigenesis. Oncogene.

[48] Huang R.S., Zheng Y.L., Zhao J., Chun X. (2018). microRNA-381 suppresses the growth and increases cisplatin sensitivity in non-small cell lung cancer cells through inhibition of nuclear factor-kappaB signaling. Biomed. Pharmacother.

[49] Wang Q., Hui H., Guo Z., Zhang W., Hu Y., He T., Tai Y., Peng P., Wang L. (2013). ADAR1 regulates ARHGAP26 gene expression through RNA editing by disrupting miR-30b-3p and miR-573 binding. RNA.

[50] Roberts J.T., Patterson D.G., King V.M., Amin S.V., Polska C.J., Houserova D., Crucello A., Barnhill E.C., Miller M.M., Sherman T.D. (2018). ADAR Mediated RNA Editing Modulates MicroRNA Targeting in Human Breast Cancer. Processes.

[51] Qi L., Song Y., Chan T.H.M., Yang H., Lin C.H., Tay D.J.T., Hong H., Tang S.J., Tan K.T., Huang X.X. (2017). An RNA editing/dsRNA binding-independent gene regulatory mechanism of ADARs and its clinical implication in cancer. Nucleic Acids Res..

[52] Nemlich Y., Greenberg E., Ortenberg R., Besser M.J., Barshack I., Jacob-Hirsch J., Jacoby E., Eyal E., Rivkin L., Prieto V.G. (2013). MicroRNA-mediated loss of ADAR1 in metastatic melanoma promotes tumor growth. J. Clin. Investig..

[53] Smith H.C., Bennett R.P., Kizilyer A., McDougall W.M., Prohaska K.M. (2012). Functions and regulation of the APOBEC family of proteins. Semin Cell Dev. Biol..

[54] Roberts S.A., Lawrence M.S., Klimczak L.J., Grimm S.A., Fargo D., Stojanov P., Kiezun A., Kryukov G.V., Carter S.L., Saksena G. (2013). An APOBEC cytidine deaminase mutagenesis pattern is widespread in human cancers. Nat. Genet..

[55] Wang S., Jia M., He Z., Liu X.S. (2018). APOBEC3B and APOBEC mutational signature as potential predictive markers for immunotherapy response in non-small cell lung cancer. Oncogene.

[56] Glaser A.P., Fantini D., Wang Y., Yu Y., Rimar K.J., Podojil J.R., Miller S.D., Meeks J.J. (2018). APOBEC-mediated mutagenesis in urothelial carcinoma is associated with improved survival, mutations in DNA damage response genes, and immune response. Oncotarget.

[57] Law E.K., Sieuwerts A.M., LaPara K., Leonard B., Starrett G.J., Molan A.M., Temiz N.A., Vogel R.I., Meijer-van Gelder M.E., Sweep F.C. (2016). The DNA cytosine deaminase APOBEC3B promotes tamoxifen resistance in ER-positive breast cancer. Sci. Adv..

[58] Blanc V., Park E., Schaefer S., Miller M., Lin Y., Kennedy S., Billing A.M., Ben Hamidane H., Graumann J., Mortazavi A. (2014). Genome-wide identification and functional analysis of Apobec-1-mediated C-to-U RNA editing in mouse small intestine and liver. Genome Biol..

[59] Salter J.D., Smith H.C. (2018). Modeling the Embrace of a Mutator: APOBEC Selection of Nucleic Acid Ligands. Trends Biochem. Sci..

[60] Yamanaka S., Balestra M.E., Ferrell L.D., Fan J., Arnold K.S., Taylor S., Taylor J.M., Innerarity T.L. (1995). Apolipoprotein B mRNA-editing protein induces hepatocellular carcinoma and dysplasia in transgenic animals. Proc. Natl. Acad. Sci. USA.

[61] Valdmanis P.N., Roy-Chaudhuri B., Kim H.K., Sayles L.C., Zheng Y., Chuang C.H., Caswell D.R., Chu K., Zhang Y., Winslow M.M. (2015). Upregulation of the microRNA cluster at the Dlk1-Dio3 locus in lung adenocarcinoma. Oncogene.

[62] Koito A., Ikeda T. (2013). Intrinsic immunity against retrotransposons by APOBEC cytidine deaminases. Front. Microbiol..

[63] Harris R.S., Liddament M.T. (2004). Retroviral restriction by APOBEC proteins. Nat. Rev. Immunol..

[64] Ding Q., Chang C.J., Xie X., Xia W., Yang J.Y., Wang S.C., Wang Y., Xia J., Chen L., Cai C. (2011). APOBEC3G promotes liver metastasis in an orthotopic mouse model of colorectal cancer and predicts human hepatic metastasis. J. Clin. Investig..

[65] Ali S., Karki N., Bhattacharya C., Zhu R., MacDuff D.A., Stenglein M.D., Schumacher A.J., Demorest Z.L., Harris R.S., Matin A. (2013). APOBEC3 inhibits DEAD-END function to regulate microRNA activity. BMC Mol. Biol..

[66] Cheng X., Chen J., Huang Z. (2018). miR-372 promotes breast cancer cell proliferation by directly targeting LATS2. Exp. Ther. Med..

[67] le Sage C., Nagel R., Egan D.A., Schrier M., Mesman E., Mangiola A., Anile C., Maira G., Mercatelli N., Ciafre S.A. (2007). Regulation of the p27(Kip1) tumor suppressor by miR-221 and miR-222 promotes cancer cell proliferation. EMBO J..

[68] Fu Y., Shao Z.M., He Q.Z., Jiang B.Q., Wu Y., Zhuang Z.G. (2015). Hsa-miR-206 represses the proliferation and invasion of breast cancer cells by targeting Cx43. Eur. Rev. Med. Pharmacol. Sci..

[69] Li M., Wang I.X., Li Y., Bruzel A., Richards A.L., Toung J.M., Cheung V.G. (2011). Widespread RNA and DNA sequence differences in the human transcriptome. Science.

[70] Pickrell J.K., Gilad Y., Pritchard J.K. (2012). Comment on “Widespread RNA and DNA sequence differences in the human transcriptome”. Science.

[71] Chakravarti A. (2011). Widespread promiscuous genetic information transfer from DNA to RNA. Circ. Res..

[72] Kleinman C.L., Majewski J. (2012). Comment on “Widespread RNA and DNA sequence differences in the human transcriptome”. Science.

[73] Wang I.X., Core L.J., Kwak H., Brady L., Bruzel A., McDaniel L., Richards A.L., Wu M., Grunseich C., Lis J.T. (2014). RNA-DNA differences are generated in human cells within seconds after RNA exits polymerase II. Cell Rep..

[74] Ling H., Spizzo R., Atlasi Y., Nicoloso M., Shimizu M., Redis R.S., Nishida N., Gafa R., Song J., Guo Z. (2013). CCAT2, a novel noncoding RNA mapping to 8q24, underlies metastatic progression and chromosomal instability in colon cancer. Genome Res..

[75] Chen B., Dragomir M.P., Fabris L., Bayraktar R., Knutsen E., Liu X., Tang C., Li Y., Shimura T., Ivkovic T.C. (2020). The Long Noncoding RNA CCAT2 induces chromosomal instability through BOP1—AURKB signaling. Gastroenterology.

[76] Haiman C.A., Le Marchand L., Yamamato J., Stram D.O., Sheng X., Kolonel L.N., Wu A.H., Reich D., Henderson B.E. (2007). A common genetic risk factor for colorectal and prostate cancer. Nat. Genet..

[77] Tomlinson I., Webb E., Carvajal-Carmona L., Broderick P., Kemp Z., Spain S., Penegar S., Chandler I., Gorman M., Wood W. (2007). A genome-wide association scan of tag SNPs identifies a susceptibility variant for colorectal cancer at 8q24.21. Nat. Genet..

[78] Zanke B.W., Greenwood C.M., Rangrej J., Kustra R., Tenesa A., Farrington S.M., Prendergast J., Olschwang S., Chiang T., Crowdy E. (2007). Genome-wide association scan identifies a colorectal cancer susceptibility locus on chromosome 8q24. Nat. Genet..

[79] Ghoussaini M., Song H., Koessler T., Al Olama A.A., Kote-Jarai Z., Driver K.E., Pooley K.A., Ramus S.J., Kjaer S.K., Hogdall E. (2008). Multiple loci with different cancer specificities within the 8q24 gene desert. J. Natl. Cancer Inst..

[80] Redis R.S., Vela L.E., Lu W., Ferreira de Oliveira J., Ivan C., Rodriguez-Aguayo C., Adamoski D., Pasculli B., Taguchi A., Chen Y. (2016). Allele-Specific Reprogramming of Cancer Metabolism by the Long Non-coding RNA CCAT2. Mol. Cell.

[81] Ontiveros R.J., Stoute J., Liu K.F. (2019). The chemical diversity of RNA modifications. Biochem. J..

[82] Hussain S., Aleksic J., Blanco S., Dietmann S., Frye M. (2013). Characterizing 5-methylcytosine in the mammalian epitranscriptome. Genome Biol..

[83] Barbieri I., Kouzarides T. (2020). Role of RNA modifications in cancer. Nat. Rev. Cancer.

[84] Dinescu S., Ignat S., Lazar A.D., Constantin C., Neagu M., Costache M. (2019). Epitranscriptomic Signatures in lncRNAs and Their Possible Roles in Cancer. Genes.

[85] Jacob R., Zander S., Gutschner T. (2017). The Dark Side of the Epitranscriptome: Chemical Modifications in Long Non-Coding RNAs. Int. J. Mol. Sci.

[86] Frye M., Watt F.M. (2006). The RNA Methyltransferase Misu (NSun2) Mediates Myc-Induced Proliferation and Is Upregulated in Tumors. Curr. Biol..

[87] Yi J., Gao R., Chen Y., Yang Z., Han P., Zhang H., Dou Y., Liu W., Wang W., Du G. (2017). Overexpression of NSUN2 by DNA hypomethylation is associated with metastatic progression in human breast cancer. Oncotarget.

[88] Trixl L., Lusser A. (2019). The dynamic RNA modification 5-methylcytosine and its emerging role as an epitranscriptomic mark. Wiley Interdiscip. Rev. RNA.

[89] Bantis A., Giannopoulos A., Gonidi M., Liossi A., Aggelonidou E., Petrakakou E., Athanassiades P., Athanassiadou P. (2004). Expression of p120, Ki-67 and PCNA as proliferation biomarkers in imprint smears of prostate carcinoma and their prognostic value. Cytopathology.

[90] Saijo Y., Sato G., Usui K., Sato M., Sagawa M., Kondo T., Minami Y., Nukiwa T. (2001). Expression of nucleolar protein p120 predicts poor prognosis in patients with stage I lung adenocarcinoma. Ann. Oncol..

[91] Jeltsch A., Ehrenhofer-Murray A., Jurkowski T.P., Lyko F., Reuter G., Ankri S., Nellen W., Schaefer M., Helm M. (2017). Mechanism and biological role of Dnmt2 in Nucleic Acid Methylation. RNA Biol..

[92] Tzelepi V., Logotheti S., Efstathiou E., Troncoso P., Aparicio A., Sakellakis M., Hoang A., Perimenis P., Melachrinou M., Logothetis C. (2020). Epigenetics and prostate cancer: Defining the timing of DNA methyltransferase deregulation during prostate cancer progression. Pathology.

[93] Yang X., Yang Y., Sun B.F., Chen Y.S., Xu J.W., Lai W.Y., Li A., Wang X., Bhattarai D.P., Xiao W. (2017). 5-methylcytosine promotes mRNA export—NSUN2 as the methyltransferase and ALYREF as an m(5)C reader. Cell Res..

[94] Fu L., Guerrero C.R., Zhong N., Amato N.J., Liu Y., Liu S., Cai Q., Ji D., Jin S.-G., Niedernhofer L.J. (2014). Tet-Mediated Formation of 5-Hydroxymethylcytosine in RNA. J. Am. Chem. Soc..

[95] Konno M., Koseki J., Asai A., Yamagata A., Shimamura T., Motooka D., Okuzaki D., Kawamoto K., Mizushima T., Eguchi H. (2019). Distinct methylation levels of mature microRNAs in gastrointestinal cancers. Nat. Commun..

[96] Cheray M., Etcheverry A., Jacques C., Pacaud R., Bougras-Cartron G., Aubry M., Denoual F., Peterlongo P., Nadaradjane A., Briand J. (2020). Cytosine methylation of mature microRNAs inhibits their functions and is associated with poor prognosis in glioblastoma multiforme. Mol. Cancer.

[97] Li Y., Li J., Luo M., Zhou C., Shi X., Yang W., Lu Z., Chen Z., Sun N., He J. (2018). Novel long noncoding RNA NMR promotes tumor progression via NSUN2 and BPTF in esophageal squamous cell carcinoma. Cancer Lett..

[98] Delatte B., Wang F., Ngoc L.V., Collignon E., Bonvin E., Deplus R., Calonne E., Hassabi B., Putmans P., Awe S. (2016). Transcriptome-wide distribution and function of RNA hydroxymethylcytosine. Science.

[99] Cimmino L., Dawlaty M.M., Ndiaye-Lobry D., Yap Y.S., Bakogianni S., Yu Y., Bhattacharyya S., Shaknovich R., Geng H., Lobry C. (2015). TET1 is a tumor suppressor of hematopoietic malignancy. Nat. Immunol..

[100] Ko M., Huang Y., Jankowska A.M., Pape U.J., Tahiliani M., Bandukwala H.S., An J., Lamperti E.D., Koh K.P., Ganetzky R. (2010). Impaired hydroxylation of 5-methylcytosine in myeloid cancers with mutant TET2. Nature.

[101] Banfai B., Jia H., Khatun J., Wood E., Risk B., Gundling W.E., Kundaje A., Gunawardena H.P., Yu Y., Xie L. (2012). Long noncoding RNAs are rarely translated in two human cell lines. Genome Res..

[102] Zhang P.F., Wei C.Y., Huang X.Y., Peng R., Yang X., Lu J.C., Zhang C., Gao C., Cai J.B., Gao P.T. (2019). Circular RNA circTRIM33-12 acts as the sponge of MicroRNA-191 to suppress hepatocellular carcinoma progression. Mol. Cancer.

[103] Hu H., Shu M., He L., Yu X., Liu X., Lu Y., Chen Y., Miao X., Chen X. (2017). Epigenomic landscape of 5-hydroxymethylcytosine reveals its transcriptional regulation of lncRNAs in colorectal cancer. Br. J. Cancer.

[104] Desrosiers R., Friderici K., Rottman F. (1974). Identification of methylated nucleosides in messenger RNA from Novikoff hepatoma cells. Proc. Natl. Acad. Sci. USA.

[105] Gilbert W.V., Bell T.A., Schaening C. (2016). Messenger RNA modifications: Form, distribution, and function. Science.

[106] Alarcon C.R., Lee H., Goodarzi H., Halberg N., Tavazoie S.F. (2015). N6-methyladenosine marks primary microRNAs for processing. Nature.

[107] Pan Y., Ma P., Liu Y., Li W., Shu Y. (2018). Multiple functions of m(6)A RNA methylation in cancer. J. Hematol. Oncol..

[108] Ping X.-L., Sun B.-F., Wang L., Xiao W., Yang X., Wang W.-J., Adhikari S., Shi Y., Lv Y., Chen Y.-S. (2014). Mammalian WTAP is a regulatory subunit of the RNA N6-methyladenosine methyltransferase. Cell Res..

[109] Cui Q., Shi H., Ye P., Li L., Qu Q., Sun G., Sun G., Lu Z., Huang Y., Yang C.-G. (2017). m^6^A RNA Methylation Regulates the Self-Renewal and Tumorigenesis of Glioblastoma Stem Cells. Cell Rep..

[110] Chen M., Wei L., Law C.-T., Tsang F.H.-C., Shen J., Cheng C.L.-H., Tsang L.-H., Ho D.W.-H., Chiu D.K.-C., Lee J.M.-F. (2018). RNA N6-methyladenosine methyltransferase-like 3 promotes liver cancer progression through YTHDF2-dependent posttranscriptional silencing of SOCS2. Hepatology.

[111] Pendleton K.E., Chen B., Liu K., Hunter O.V., Xie Y., Tu B.P., Conrad N.K. (2017). The U6 snRNA m(6)A Methyltransferase METTL16 Regulates SAM Synthetase Intron Retention. Cell.

[112] Sun Y., Ma L. (2019). New Insights into Long Non-Coding RNA MALAT1 in Cancer and Metastasis. Cancers.

[113] Brown J.A., Kinzig C.G., DeGregorio S.J., Steitz J.A. (2016). Methyltransferase-like protein 16 binds the 3′-terminal triple helix of MALAT1 long noncoding RNA. Proc. Natl. Acad. Sci. USA.

[114] McCown P.J., Wang M.C., Jaeger L., Brown J.A. (2019). Secondary Structural Model of Human MALAT1 Reveals Multiple Structure-Function Relationships. Int. J. Mol. Sci..

[115] Patil D.P., Chen C.-K., Pickering B.F., Chow A., Jackson C., Guttman M., Jaffrey S.R. (2016). m6A RNA methylation promotes XIST-mediated transcriptional repression. Nature.

[116] Chen T., Hao Y.-J., Zhang Y., Li M.-M., Wang M., Han W., Wu Y., Lv Y., Hao J., Wang L. (2015). m^6^A RNA Methylation Is Regulated by MicroRNAs and Promotes Reprogramming to Pluripotency. Cell Stem Cell.

[117] Meyer K.D., Jaffrey S.R. (2017). Rethinking m(6)A Readers, Writers, and Erasers. Annu. Rev. Cell Dev. Biol..

[118] Bai Y., Yang C., Wu R., Huang L., Song S., Li W., Yan P., Lin C., Li D., Zhang Y. (2019). YTHDF1 Regulates Tumorigenicity and Cancer Stem Cell-Like Activity in Human Colorectal Carcinoma. Front. Oncol..

[119] Wang X., Lu Z., Gomez A., Hon G.C., Yue Y., Han D., Fu Y., Parisien M., Dai Q., Jia G. (2014). N6-methyladenosine-dependent regulation of messenger RNA stability. Nature.

[120] Paris J., Morgan M., Campos J., Spencer G.J., Shmakova A., Ivanova I., Mapperley C., Lawson H., Wotherspoon D.A., Sepulveda C. (2019). Targeting the RNA m^6^ A Reader YTHDF2 Selectively Compromises Cancer Stem Cells in Acute Myeloid Leukemia. Cell Stem Cell.

[121] Tanabe A., Tanikawa K., Tsunetomi M., Takai K., Ikeda H., Konno J., Torigoe T., Maeda H., Kutomi G., Okita K. (2016). RNA helicase YTHDC2 promotes cancer metastasis via the enhancement of the efficiency by which HIF-1alpha mRNA is translated. Cancer Lett..

[122] Alarcón Claudio R., Goodarzi H., Lee H., Liu X., Tavazoie S., Tavazoie Sohail F. (2015). HNRNPA2B1 Is a Mediator of m^6^ A-Dependent Nuclear RNA Processing Events. Cell.

[123] Jia G., Fu Y., Zhao X., Dai Q., Zheng G., Yang Y., Yi C., Lindahl T., Pan T., Yang Y.G. (2011). N6-methyladenosine in nuclear RNA is a major substrate of the obesity-associated FTO. Nat. Chem. Biol..

[124] Zheng G., Dahl J.A., Niu Y., Fedorcsak P., Huang C.-M., Li C.J., Vågbø Cathrine B., Shi Y., Wang W.-L., Song S.-H. (2013). ALKBH5 Is a Mammalian RNA Demethylase that Impacts RNA Metabolism and Mouse Fertility. Mol. Cell.

[125] Li Z., Weng H., Su R., Weng X., Zuo Z., Li C., Huang H., Nachtergaele S., Dong L., Hu C. (2017). FTO Plays an Oncogenic Role in Acute Myeloid Leukemia as a N^6^-Methyladenosine RNA Demethylase. Cancer Cell.

[126] Yang S., Wei J., Cui Y.-H., Park G., Shah P., Deng Y., Aplin A.E., Lu Z., Hwang S., He C. (2019). m6A mRNA demethylase FTO regulates melanoma tumorigenicity and response to anti-PD-1 blockade. Nat. Commun..

[127] Zhang S., Zhao B.S., Zhou A., Lin K., Zheng S., Lu Z., Chen Y., Sulman E.P., Xie K., Bögler O. (2017). m^6^ A Demethylase ALKBH5 Maintains Tumorigenicity of Glioblastoma Stem-like Cells by Sustaining FOXM1 Expression and Cell Proliferation Program. Cancer Cell.

[128] Zhang C., Samanta D., Lu H., Bullen J.W., Zhang H., Chen I., He X., Semenza G.L. (2016). Hypoxia induces the breast cancer stem cell phenotype by HIF-dependent and ALKBH5-mediated m⁶A-demethylation of NANOG mRNA. Proc. Natl. Acad. Sci. USA.

[129] Saikia M., Fu Y., Pavon-Eternod M., He C., Pan T. (2010). Genome-wide analysis of N1-methyl-adenosine modification in human tRNAs. RNA.

[130] Li X., Xiong X., Wang K., Wang L., Shu X., Ma S., Yi C. (2016). Transcriptome-wide mapping reveals reversible and dynamic N1-methyladenosine methylome. Nat. Chem. Biol..

[131] Dominissini D., Nachtergaele S., Moshitch-Moshkovitz S., Peer E., Kol N., Ben-Haim M.S., Dai Q., Di Segni A., Salmon-Divon M., Clark W.C. (2016). The dynamic N1-methyladenosine methylome in eukaryotic messenger RNA. Nature.

[132] Zhou H., Rauch S., Dai Q., Cui X., Zhang Z., Nachtergaele S., Sepich C., He C., Dickinson B.C. (2019). Evolution of a reverse transcriptase to map N(1)-methyladenosine in human messenger RNA. Nat. Methods.

[133] Dai X., Wang T., Gonzalez G., Wang Y. (2018). Identification of YTH Domain-Containing Proteins as the Readers for N1-Methyladenosine in RNA. Anal. Chem..

[134] Gutschner T., Hämmerle M., Diederichs S. (2013). MALAT1—A paradigm for long noncoding RNA function in cancer. J. Mol. Med..

[135] Xiong X., Li X., Yi C. (2018). N1-methyladenosine methylome in messenger RNA and non-coding RNA. Curr. Opin. Chem. Biol..

[136] Li X., Xiong X., Zhang M., Wang K., Chen Y., Zhou J., Mao Y., Lv J., Yi D., Chen X.-W. (2017). Base-Resolution Mapping Reveals Distinct m^1^ A Methylome in Nuclear- and Mitochondrial-Encoded Transcripts. Mol. Cell.

[137] Safra M., Sas-Chen A., Nir R., Winkler R., Nachshon A., Bar-Yaacov D., Erlacher M., Rossmanith W., Stern-Ginossar N., Schwartz S. (2017). The m1A landscape on cytosolic and mitochondrial mRNA at single-base resolution. Nature.

[138] Liu F., Clark W., Luo G., Wang X., Fu Y., Wei J., Wang X., Hao Z., Dai Q., Zheng G. (2016). ALKBH1-Mediated tRNA Demethylation Regulates Translation. Cell.

[139] Chen Z., Qi M., Shen B., Luo G., Wu Y., Li J., Lu Z., Zheng Z., Dai Q., Wang H. (2019). Transfer RNA demethylase ALKBH3 promotes cancer progression via induction of tRNA-derived small RNAs. Nucleic Acids Res..

[140] Woo H.-H., Chambers S.K. (2019). Human ALKBH3-induced m1A demethylation increases the CSF-1 mRNA stability in breast and ovarian cancer cells. Biochim. Biophys. Acta BBA Gene Regul. Mech..

[141] Yamato I., Sho M., Shimada K., Hotta K., Ueda Y., Yasuda S., Shigi N., Konishi N., Tsujikawa K., Nakajima Y. (2012). PCA-1/ALKBH3 Contributes to Pancreatic Cancer by Supporting Apoptotic Resistance and Angiogenesis. Cancer Res..

[142] Cohn W.E., Volkin E. (1951). Nucleoside-5′-Phosphates from Ribonucleic Acid. Nature.

[143] Ge J., Yu Y.-T. (2013). RNA pseudouridylation: New insights into an old modification. Trends Biochem. Sci..

[144] Karijolich J., Yi C., Yu Y.-T. (2015). Transcriptome-wide dynamics of RNA pseudouridylation. Nat. Rev. Mol. Cell Biol..

[145] Balogh E., Chandler J.C., Varga M., Tahoun M., Menyhard D.K., Schay G., Goncalves T., Hamar R., Legradi R., Szekeres A. (2020). Pseudouridylation defect due to DKC1 and NOP10 mutations causes nephrotic syndrome with cataracts, hearing impairment, and enterocolitis. Proc. Natl. Acad. Sci. USA.

[146] Penzo M., Ludovini V., Treré D., Siggillino A., Vannucci J., Bellezza G., Crinò L., Montanaro L. (2015). Dyskerin and TERC expression may condition survival in lung cancer patients. Oncotarget.

[147] Sieron P., Hader C., Hatina J., Engers R., Wlazlinski A., Müller M., Schulz W.A. (2009). DKC1 overexpression associated with prostate cancer progression. Br. J. Cancer.

[148] Montanaro L., Calienni M., Bertoni S., Rocchi L., Sansone P., Storci G., Santini D., Ceccarelli C., Taffurelli M., Carnicelli D. (2010). Novel Dyskerin-Mediated Mechanism of p53 Inactivation through Defective mRNA Translation. Cancer Res..

[149] Huang H., Weng H., Deng X., Chen J. (2020). RNA Modifications in Cancer: Functions, Mechanisms, and Therapeutic Implications. Annu. Rev. Cancer Biol..

[150] Zhao X., Patton J.R., Davis S.L., Florence B., Ames S.J., Spanjaard R.A. (2004). Regulation of Nuclear Receptor Activity by a Pseudouridine Synthase through Posttranscriptional Modification of Steroid Receptor RNA Activator. Mol. Cell.

[151] Jana S., Hsieh A.C., Gupta R. (2017). Reciprocal amplification of caspase-3 activity by nuclear export of a putative human RNA-modifying protein, PUS10 during TRAIL-induced apoptosis. Cell Death Dis..

[152] Li X., Zhu P., Ma S., Song J., Bai J., Sun F., Yi C. (2015). Chemical pulldown reveals dynamic pseudouridylation of the mammalian transcriptome. Nat. Chem. Biol..

[153] Schwartz S., Bernstein D.A., Mumbach M.R., Jovanovic M., Herbst R.H., León-Ricardo B.X., Engreitz J.M., Guttman M., Satija R., Lander E.S. (2014). Transcriptome-wide Mapping Reveals Widespread Dynamic-Regulated Pseudouridylation of ncRNA and mRNA. Cell.

[154] Yang H., Li G., Cheng B., Jiang R. (2018). ZFAS1 functions as an oncogenic long non-coding RNA in bladder cancer. Biosci. Rep..

[155] Liu F., Gao H., Li S., Ni X., Zhu Z. (2017). Long non-coding RNA ZFAS1 correlates with clinical progression and prognosis in cancer patients. Oncotarget.

[156] Grammatikakis I., Panda A.C., Abdelmohsen K., Gorospe M. (2014). Long noncoding RNAs(lncRNAs) and the molecular hallmarks of aging. Aging.

[157] Baena-Del Valle J.A., Zheng Q., Esopi D.M., Rubenstein M., Hubbard G.K., Moncaliano M.C., Hruszkewycz A., Vaghasia A., Yegnasubramanian S., Wheelan S.J. (2018). MYC drives overexpression of telomerase RNA (hTR/TERC) in prostate cancer. J. Pathol..

[158] Carlile T.M., Rojas-Duran M.F., Zinshteyn B., Shin H., Bartoli K.M., Gilbert W.V. (2014). Pseudouridine profiling reveals regulated mRNA pseudouridylation in yeast and human cells. Nature.

[159] Wang M.W., Liu J., Liu Q., Xu Q.H., Li T.F., Jin S., Xia T.S. (2017). LncRNA SNHG7 promotes the proliferation and inhibits apoptosis of gastric cancer cells by repressing the P15 and P16 expression. Eur. Rev. Med. Pharmacol. Sci..

[160] Tian T., Qiu R., Qiu X. (2018). SNHG1 promotes cell proliferation by acting as a sponge of miR-145 in colorectal cancer. Oncotarget.

[161] Liu L., Shi Y., Shi J., Wang H., Sheng Y., Jiang Q., Chen H., Li X., Dong J. (2019). The long non-coding RNA SNHG1 promotes glioma progression by competitively binding to miR-194 to regulate PHLDA1 expression. Cell Death Dis..

[162] Menezes M.R., Balzeau J., Hagan J.P. (2018). 3′ RNA Uridylation in Epitranscriptomics, Gene Regulation, and Disease. Front. Mol. Biosci..

[163] Zhu D.-Q., Lou Y.-F., He Z.-G., Ji M. (2014). Nucleotidyl transferase TUT1 inhibits lipogenesis in osteosarcoma cells through regulation of microRNA-24 and microRNA-29a. Tumor Biol..

[164] Yu C., Gong Y., Zhou H., Wang M., Kong L., Liu J., An T., Zhu H., Li Y. (2017). Star-PAP, a poly(A) polymerase, functions as a tumor suppressor in an orthotopic human breast cancer model. Cell Death Dis..

[165] Alajez N.M., Shi W., Wong D., Lenarduzzi M., Waldron J., Weinreb I., Liu F.F. (2012). Lin28b promotes head and neck cancer progression via modulation of the insulin-like growth factor survival pathway. Oncotarget.

[166] Cai W.Y., Wei T.Z., Luo Q.C., Wu Q.W., Liu Q.F., Yang M., Ye G.D., Wu J.F., Chen Y.Y., Sun G.B. (2013). The Wnt-beta-catenin pathway represses let-7 microRNA expression through transactivation of Lin28 to augment breast cancer stem cell expansion. J. Cell Sci..

[167] Thornton J.E., Chang H.-M., Piskounova E., Gregory R.I. (2012). Lin28-mediated control of let-7 microRNA expression by alternative TUTases Zcchc11 (TUT4) and Zcchc6 (TUT7). RNA.

[168] Heo I., Ha M., Lim J., Yoon M.-J., Park J.-E., Kwon S.C., Chang H., Kim V.N. (2012). Mono-Uridylation of Pre-MicroRNA as a Key Step in the Biogenesis of Group II let-7 MicroRNAs. Cell.

[169] Ustianenko D., Hrossova D., Potesil D., Chalupnikova K., Hrazdilova K., Pachernik J., Cetkovska K., Uldrijan S., Zdrahal Z., Vanacova S. (2013). Mammalian DIS3L2 exoribonuclease targets the uridylated precursors of let-7 miRNAs. RNA.

[170] Hunter R.W., Liu Y., Manjunath H., Acharya A., Jones B.T., Zhang H., Chen B., Ramalingam H., Hammer R.E., Xie Y. (2018). Loss of Dis3l2 partially phenocopies Perlman syndrome in mice and results in up-regulation of Igf2 in nephron progenitor cells. Genes Dev..

[171] Pandolfini L., Barbieri I., Bannister A.J., Hendrick A., Andrews B., Webster N., Murat P., Mach P., Brandi R., Robson S.C. (2019). METTL1 Promotes let-7 MicroRNA Processing via m7G Methylation. Mol. Cell.

[172] Okamoto M., Fujiwara M., Hori M., Okada K., Yazama F., Konishi H., Xiao Y., Qi G., Shimamoto F., Ota T. (2014). tRNA modifying enzymes, NSUN2 and METTL1, determine sensitivity to 5-fluorouracil in HeLa cells. PLoS Genet..

[173] Gustavsson M., Ronne H. (2008). Evidence that tRNA modifying enzymes are important in vivo targets for 5-fluorouracil in yeast. RNA.

[174] Cowling V.H. (2010). Enhanced mRNA cap methylation increases cyclin D1 expression and promotes cell transformation. Oncogene.

[175] Vasilescu C., Dragomir M., Tanase M., Giza D., Purnichescu-Purtan R., Chen M., Yeung S.J., Calin G.A. (2017). Circulating miRNAs in sepsis-A network under attack: An in-silico prediction of the potential existence of miRNA sponges in sepsis. PLoS ONE.

[176] Dragomir M., Mafra A.C.P., Dias S.M.G., Vasilescu C., Calin G.A. (2018). Using microRNA Networks to Understand Cancer. Int. J. Mol. Sci..

[177] Van Roosbroeck K., Bayraktar R., Calin S., Bloehdorn J., Dragomir M.P., Okubo K., Bertilaccio M.T.S., Zupo S., You M.J., Gaidano G. (2019). The involvement of microRNA in the pathogenesis of Richter syndrome. Haematologica.

[178] Fuentes-Mattei E., Bayraktar R., Manshouri T., Silva A.M., Ivan C., Gulei D., Fabris L., Soares do Amaral N., Mur P., Perez C. (2020). miR-543 regulates the epigenetic landscape of myelofibrosis by targeting TET1 and TET2. JCI Insight.

[179] Volinia S., Galasso M., Costinean S., Tagliavini L., Gamberoni G., Drusco A., Marchesini J., Mascellani N., Sana M.E., Abu Jarour R. (2010). Reprogramming of miRNA networks in cancer and leukemia. Genome Res..

[180] Sieuwerts A.M., Willis S., Burns M.B., Look M.P., Meijer-Van Gelder M.E., Schlicker A., Heideman M.R., Jacobs H., Wessels L., Leyland-Jones B. (2014). Elevated APOBEC3B correlates with poor outcomes for estrogen-receptor-positive breast cancers. Horm. Cancer.

[181] Okugawa Y., Toiyama Y., Shigeyasu K., Yamamoto A., Shigemori T., Yin C., Ichikawa T., Yasuda H., Fujikawa H., Yoshiyama S. (2018). Enhanced AZIN1 RNA editing and overexpression of its regulatory enzyme ADAR1 are important prognostic biomarkers in gastric cancer. J. Transl. Med..

[182] Chan T.H., Qamra A., Tan K.T., Guo J., Yang H., Qi L., Lin J.S., Ng V.H., Song Y., Hong H. (2016). ADAR-Mediated RNA Editing Predicts Progression and Prognosis of Gastric Cancer. Gastroenterology.

[183] Liu G.M., Zeng H.D., Zhang C.Y., Xu J.W. (2020). Identification of METTL3 as an Adverse Prognostic Biomarker in Hepatocellular Carcinoma. Dig. Dis. Sci..

[184] Niu Y., Lin Z., Wan A., Chen H., Liang H., Sun L., Wang Y., Li X., Xiong X.F., Wei B. (2019). RNA N6-methyladenosine demethylase FTO promotes breast tumor progression through inhibiting BNIP3. Mol. Cancer.

[185] Ni W., Yao S., Zhou Y., Liu Y., Huang P., Zhou A., Liu J., Che L., Li J. (2019). Long noncoding RNA GAS5 inhibits progression of colorectal cancer by interacting with and triggering YAP phosphorylation and degradation and is negatively regulated by the m^6^ A reader YTHDF3. Mol. Cancer.

[186] Petrescu G.E.D., Sabo A.A., Torsin L.I., Calin G.A., Dragomir M.P. (2019). MicroRNA based theranostics for brain cancer: Basic principles. J. Exp. Clin. Cancer Res..

[187] Shah M.Y., Ferrajoli A., Sood A.K., Lopez-Berestein G., Calin G.A. (2016). microRNA Therapeutics in Cancer—An Emerging Concept. EBioMedicine.

[188] Pichler M., Rodriguez-Aguayo C., Nam S.Y., Dragomir M.P., Bayraktar R., Anfossi S., Knutsen E., Ivan C., Fuentes-Mattei E., Lee S.K. (2020). Therapeutic potential of FLANC, a novel primate-specific long non-coding RNA in colorectal cancer. Gut.

[189] Selvam C., Mutisya D., Prakash S., Ranganna K., Thilagavathi R. (2017). Therapeutic potential of chemically modified siRNA: Recent trends. Chem. Biol. Drug Des..

[190] Boriack-Sjodin P.A., Ribich S., Copeland R.A. (2018). RNA-modifying proteins as anticancer drug targets. Nat. Rev. Drug Discov..

[191] Li T., Hu P.S., Zuo Z., Lin J.F., Li X., Wu Q.N., Chen Z.H., Zeng Z.L., Wang F., Zheng J. (2019). METTL3 facilitates tumor progression via an m(6)A-IGF2BP2-dependent mechanism in colorectal carcinoma. Mol. Cancer.

[192] Bedi R.K., Huang D., Eberle S.A., Wiedmer L., Sledz P., Caflisch A. (2020). Small-Molecule Inhibitors of METTL3, the Major Human Epitranscriptomic Writer. Chem. Med. Chem..

[193] Schapira M., Calabrese M.F., Bullock A.N., Crews C.M. (2019). Targeted protein degradation: Expanding the toolbox. Nat. Rev. Drug Discov..

[194] Xiao L., Li X., Mu Z., Zhou J., Zhou P., Xie C., Jiang S. (2020). FTO Inhibition Enhances the Antitumor Effect of Temozolomide by Targeting MYC-miR-155/23a Cluster-MXI1 Feedback Circuit in Glioma. Cancer Res..

[195] Su R., Dong L., Li Y., Gao M., Han L., Wunderlich M., Deng X., Li H., Huang Y., Gao L. (2020). Targeting FTO Suppresses Cancer Stem Cell Maintenance and Immune Evasion. Cancer Cell.

[196] Huang Y., Su R., Sheng Y., Dong L., Dong Z., Xu H., Ni T., Zhang Z.S., Zhang T., Li C. (2019). Small-Molecule Targeting of Oncogenic FTO Demethylase in Acute Myeloid Leukemia. Cancer Cell.

